# EHR-Safe: generating high-fidelity and privacy-preserving synthetic electronic health records

**DOI:** 10.1038/s41746-023-00888-7

**Published:** 2023-08-11

**Authors:** Jinsung Yoon, Michel Mizrahi, Nahid Farhady Ghalaty, Thomas Jarvinen, Ashwin S. Ravi, Peter Brune, Fanyu Kong, Dave Anderson, George Lee, Arie Meir, Farhana Bandukwala, Elli Kanal, Sercan Ö. Arık, Tomas Pfister

**Affiliations:** 1 Google Cloud, 1155 Borregas Ave, Sunnyvale, CA USA; 2 Google LLC, 1600 Amphitheatre Pkwy, Mountain View, CA USA

**Keywords:** Translational research, Medical research

## Abstract

Privacy concerns often arise as the key bottleneck for the sharing of data between consumers and data holders, particularly for sensitive data such as Electronic Health Records (EHR). This impedes the application of data analytics and ML-based innovations with tremendous potential. One promising approach for such privacy concerns is to instead use synthetic data. We propose a generative modeling framework, EHR-Safe, for generating highly realistic and privacy-preserving synthetic EHR data. EHR-Safe is based on a two-stage model that consists of sequential encoder-decoder networks and generative adversarial networks. Our innovations focus on the key challenging aspects of real-world EHR data: heterogeneity, sparsity, coexistence of numerical and categorical features with distinct characteristics, and time-varying features with highly-varying sequence lengths. Under numerous evaluations, we demonstrate that the fidelity of EHR-Safe is almost-identical with real data (<3% accuracy difference for the models trained on them) while yielding almost-ideal performance in practical privacy metrics.

## Introduction

Electronic Health Records (EHR) provide tremendous potential for enhancing patient care, embedding performance measures in clinical practice, and facilitating clinical research. Statistical estimation and machine learning models trained on EHR data can be used to diagnose diseases (such as diabetes^1^, track patient wellness^2^, and predict how patients respond to specific drugs^3^). To develop such models, researchers and practitioners need access to data. However, data privacy concerns and patient confidentiality regulations continue to pose a major barrier to data access^4–6^.

Conventional methods to anonymize data can be tedious and costly^7,8^. They can distort important features from the original dataset, decreasing the utility of the data significantly, and they can be susceptible to privacy attacks even when the de-identification process is in accordance with existing standards^9^. Synthetic data open new horizons for data sharing^10^. With two key properties, synthetic data can be extremely useful: (1) high fidelity (i.e., the synthesized data are useful for the task of interest, such as giving similar downstream performance when a diagnostic model is trained on them), (2) meets certain privacy measures (i.e., the synthesized data do not reveal any real patient’s identity).

Generative models have shown notable success in generating synthetic data^11–15^. They are trained to synthesize data from a given random noise vector or a feature that the model is conditioned on. This comes with the premise, for privacy preservation, that the data samples synthesized from random vectors should be distinct from the real ones. Among generative models, Generative Adversarial Networks (GANs)^16^ have particularly gained traction as they can synthesize highly realistic samples from the actual distribution of real data. The notable success of GANs in synthesizing high-dimensional complex data has been shown for images^17^, speech^18^, text^19^ and time-series^15^. Recent works have also adapted GANs for privacy-preserving data generation, with methods such as adding noise to model weights^20^ or modified adversarial training^21^.

When it comes to synthetic EHR data generation, there are multiple fundamental challenges. EHR data contain heterogeneous features with different characteristics and distributions. There can be numerical features (e.g., blood pressure) as well as categorical features, with many (e.g., medical codes) or two (e.g., mortality outcome) categories. We note that EHR data with images and free-form text are beyond the scope of this paper. Some of these features might be static (i.e., not varying during the modeling window), while others are time-varying, such as regular or sporadic lab measurements or diagnoses. Feature distributions might come from quite different families—categorical distributions might be highly nonuniform (e.g., if there are minority groups), and numerical distributions might be highly skewed (e.g., a small proportion of values being very large while the vast majority are small). Ideally, a generative model should have sufficient capacity to model all these types of features. Depending on a patient’s condition, the number of visits might vary drastically—some patients might visit a clinic only once, whereas some might visit hundreds of times, leading to a variance in sequence lengths that is typically much higher compared to other time-series data. There might also be a high ratio of missing features across different patients and time steps, as not all lab measurements or other input data might have been collected. An effective generative model should be realistic in synthesizing missing patterns.

GANs have been extended to healthcare data, particularly for EHR.^22–24^ apply various GAN variants on EHR data. However, these variants have limitations regarding the aforementioned fundamental aspects of real-world EHR data, such as dealing with missing features, varying feature length (rather than fixed length), categorical features (beyond numerical), and static features (beyond time series). These fundamental challenges require a holistic re-design in GAN-based synthetic data generation systems. In this paper, our goal is to push the state-of-the-art by designing a framework that can jointly represent these diverse data modalities while preserving the privacy of source training data.

**Fig. 1:**
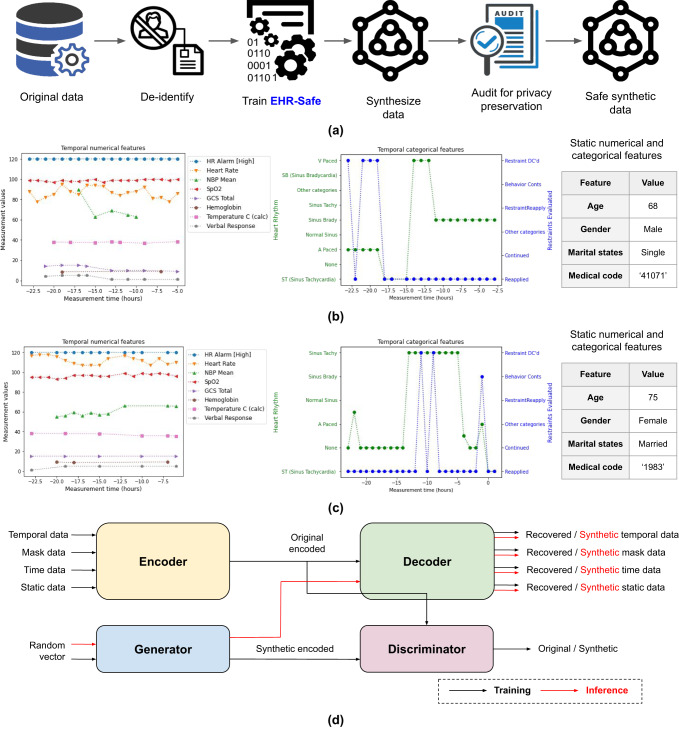
Proposed EHR-Safe framework. **a** Overall flowchart of generating synthetic data from the original data. In this paper, we mainly focus on training EHR-Safe and synthesizing synthetic data. **b** Example of real EHR data containing static and temporal features with numerical and categorical values. **c** Example of synthetic EHR data containing static and temporal features with numerical and categorical values. **d** Overall block diagram of EHR-Safe. At inference, we only use the trained generator and decoder to generate synthetic data (followed by the red arrows).

EHR-Safe, overviewed in Fig. 1, generates synthetic data that maintain the relevant statistical properties of the downstream tasks while preserving the privacy of the original data. Our methodological innovations are key to this—we introduce approaches for encoding/decoding features, normalizing complex distributions, conditioning adversarial training, and representing missing data. We demonstrate our results on two large-scale real-world EHR datasets: MIMIC-III^25–27^ and eICU^28^. We demonstrate superior synthetic data generation on a range of fidelity and privacy metrics, often outperforming the previous works by a large margin.

## Results

### Datasets

We utilize two real-world de-identified EHR datasets to showcase the EHR-Safe framework: (1) MIMIC-III (https://physionet.org/content/mimiciii/1.4/), (2) eICU (https://eicu-crd.mit.edu/gettingstarted/access/). Both are inpatient datasets that consist of varying lengths of sequences and include multiple static and temporal features with missing components.

#### MIMIC-III

The total number of patients is 19,946. Among more than 3000 features, we select 90 heterogeneous features that have high correlations with the mortality outcome (Details can be found in Supplementary Information). Ninety features consist of (1) 3 static numerical features (e.g., age), (2) 3 static categorical features (e.g., marital status), (3) 75 temporal numerical features (e.g., respiratory rate), (4) 8 temporal categorical features (e.g., heart rhythm), and (5) 1 measurement time. The sequence lengths vary between 1 and 30.

#### eICU

The total number of patients is 198,707. There are (1) 3 static numerical features (age, gender, mortality), (2) 1 static categorical feature (condition code), (3) 162 temporal numerical features, and (4) 1 measurement time. Among 162 temporal numerical features, we only select 50 features whose average number of observations is higher than 1 per patient. We set the maximum length of sequence as 50. For longer sequences, we only use the last 50 time steps.

For both datasets, we divide the patients into disjoint train and test datasets with 80 and 20% ratios. We only use the training split to train EHR-Safe. At inference, we generate synthetic train and test datasets from random vectors (note that EHR-Safe can generate an arbitrary amount of synthetic samples). We apply standard outlier removal methods (by removing the sample whose values are outside of certain value ranges between 0.1 percentile and 99.9 percentile) to exclude the outliers from the original datasets. More details on datasets, training and evaluation can be found in Supplementary Information.

### Fidelity

The fidelity metrics assess the quality of synthetically generated data by measuring the realisticness of the synthetic data compared with real data (more details are provided in Supplementary Information). Higher fidelity implies that it is more difficult to differentiate between synthetic and real data. For generative modeling, there is no standard way of evaluating the fidelity of the generated synthetic data samples, and often different works base their evaluations on different methods. In this section, we evaluate the fidelity of synthetic data with multiple quantitative and qualitative analyses, including training on synthetic/testing on real and KS-statistics. More results (including t-SNE analyses, comparison of distributions, propensity scores, and feature importance) can be found in Supplementary Information.

#### Statistical similarity

We provide quantitative comparisons of statistical similarity between original and synthetic data that compare the distributions of the generated synthetic data and original data per each feature (including the missing patterns). For numeric variables, we report the mean, standard deviation, missing rates, and KS-statistics. For categorical data, we report the ratio of each category. We only report the results with the 15 temporal numerical features (with lowest missing rates) and all static numerical features. Table 1 summarizes the results for temporal and static numerical features, and most statistics are well-aligned between original and synthetic data (KS-statistics are mostly lower than 0.03). Additional results of the top 50 temporal numerical features and categorical features can be found in Supplementary Information.

**Table 1:** Statistical similarity analyses.

MIMIC-III Dataset								
Feature type	Feature name	Original data			Synthetic data			KS-Stats
		Mean	Std	Miss rate (%)	Mean	Std	Miss rate (%)	
Temporal	Heart rate	82.56	17.34	36.53	82.20	15.91	35.74	0.01
	Respiratory rate	18.85	5.31	37.88	18.29	4.54	36.72	0.04
	calprevflg	1.00	0.00	66.68	1.00	0.00	66.65	0.00
	SpO_2_	97.30	3.38	67.99	97.39	2.27	67.39	0.03
	O_2_ saturation pulse oximetry	96.89	3.12	70.63	96.97	2.41	70.00	0.02
	NBP [systolic]	119.86	22.78	78.24	117.53	19.77	79.20	0.04
	NBP [diastolic]	56.64	14.75	78.26	56.89	13.20	79.29	0.03
	NBP mean	76.01	14.82	78.60	75.16	13.51	79.90	0.03
	HR alarm [low]	54.21	8.39	79.43	53.98	5.13	79.43	0.02
	HR alarm [high]	120.28	11.86	79.48	120.15	8.75	79.44	0.01
	SpO_2_ alarm [low]	89.73	8.77	33.76	89.54	9.43	33.61	0.02
	SpO_2_ alarm [high]	99.14	7.67	36.53	99.37	6.52	35.74	0.00
	Resp alarm [high]	32.77	8.14	37.88	32.17	5.34	36.72	0.03
	Resp alarm [low]	8.78	7.57	66.68	8.61	6.64	66.65	0.00
	Previous weight (F)	77.70	21.82	67.99	77.97	17.19	67.39	0.06
Static	Age	91.33	67.41	0.00	93.05	70.15	0.00	0.02
	Gender	0.51	0.49	0.00	0.52	0.49	0.00	0.00
	Mortality	0.10	0.30	0.00	0.09	0.29	0.00	0.01

eICU Dataset								
Feature type	Feature name	Original data			Synthetic data			KS-Stats
		Mean	Std	Miss rate (%)	Mean	Std	Miss rate (%)	
Temporal	Noninvasive mean	81.65	16.48	50.47	82.39	15.16	48.61	0.03
	Noninvasive systolic	121.97	22.62	50.57	121.79	20.60	48.62	0.02
	Noninvasive diastolic	65.34	14.59	50.57	65.80	13.02	48.67	0.03
	Bedside glucose	150.86	59.10	81.44	149.28	49.85	84.62	0.04
	Potassium	3.98	0.55	91.02	3.92	0.48	91.98	0.04
	Hgb	10.35	2.14	91.98	10.47	2.10	92.17	0.04
	Glucose	130.45	48.72	91.98	132.15	47.56	92.26	0.03
	Ssodium	138.01	4.98	91.66	138.26	4.36	92.37	0.02
	Creatinine	1.35	1.20	92.07	1.34	1.11	92.42	0.01
	Hct	31.49	6.19	92.10	31.76	6.06	92.43	0.03
	BUN	24.37	17.55	92.12	23.23	16.67	92.89	0.04
	Calcium	8.42	0.71	92.43	8.39	0.70	92.66	0.03
	Bicarbonate	25.44	4.81	92.46	25.21	4.31	93.02	0.03
	Platelets x 1000	215.19	104.12	92.74	207.75	94.41	93.30	0.02
	WBC x 1000	10.39	4.83	92.81	10.00	4.16	93.53	0.02
Static	Age	63.05	17.07	0.00	64.25	16.82	0.00	0.03
	Gender	0.54	0.49	0.00	0.54	0.49	0.00	0.00
	Mortality	0.049	0.21	0.00	0.048	0.21	0.00	0.00

Analyses on numerical temporal and static features of MIMIC-III and eICU data. KS-stats represent the maximum cumulative distribution function (CDF) difference between original and synthetic features (we ignore missing components when computing KS-stats).

#### Utility—ML model development on synthetic vs. real data

As one of the most important use cases of synthetic data is enabling machine learning innovations, we focus on the fidelity metric that compares a predictive model performance when it is trained on synthetic vs. real data. Similar model performance would indicate that the synthetic data captures the relevant informative content for the task.

We focus on the mortality prediction task^29,30^, one of the most important machine learning tasks for EHR. We train four different predictive models (Gradient Boosting Tree Ensemble (GBDT), Random Forest (RF), Logistic Regression (LR), Gated Recurrent Units (GRU)). Table 2 compares the performance of the predictive models. In most scenarios, they are highly similar in terms of AUC. On MIMIC-III, the best model (GBDT) on synthetic data is only 0.026 worse than the best model on real data, whereas on eICU, the best model (RF) on synthetic data is only 0.009 worse than the best model on real data. In Supplementary Information, we also provide the algorithmic fairness analysis across multiple subgroups divided by static categorical features (such as gender and religion).

**Table 2:** Fidelity results with utility metrics.

Utility with all features						
Target	Models	Metrics	MIMIC-III		eICU	
			Train on Real	Train on Synth	Train on Real	Train on Synth
Mortality	GBDT	AUC	**0.762**	**0.736**	0.943	0.938
		AP	**0.304**	**0.261**	0.600	0.534
	RF	AUC	0.723	0.710	**0.954**	**0.945**
		AP	0.276	0.251	**0.600**	**0.580**
	GRU	AUC	0.728	0.667	0.937	0.938
		AP	0.278	0.193	0.567	0.528
	LR	AUC	0.712	0.680	0.872	0.818
		AP	0.233	0.207	0.323	0.260
	Average	AUC	0.731	0.689	0.926	0.909
		AP	0.272	0.228	0.522	0.475

Utility with random subsets of features						
Target	Models	Metrics	MIMIC-III		eICU	
			Mean-diff	*p*-value (*X* = 0.04)	Mean-diff	*p*-value (*X* = 0.04)
Mortality	RF	AUC	0.009	0.000	0.009	0.000
		AP	0.035	0.000	0.035	0.098
Gender		AUC	0.065	1.000	0.019	0.000
		AP	0.046	0.860	0.013	0.000

(Upper) Downstream task performance with four different predictive models and two different settings (train on real vs. train on synthetic) on MIMIC-III and eICU datasets. Performance is evaluated on the original test sets. The best performance in each column is shown in bold. (Lower) The average absolute performance difference (in terms of AUC/AP) between training on real vs. synthetic data and the corresponding *p*-values (computed by one sample T-test) for predicting mortality and gender with random subsets of features.

Additionally, we evaluate the utility of the synthetic data with a random subset of features and multiple target variables. The goal is to evaluate the predictive capability of each dataset regardless of which features and targets are being used. We choose random subsets with 30 features and two target variables (mortality and gender) and test the hypothesis that the performance difference between the trained models by original and synthetic data is greater than *X*. In a practical setting, the choice of X would enable data owners to define a constraint on the acceptable fidelity of synthetic data. We report results with *X* = 0.04 for illustrative purposes. We obtain the *p*-value (computed by one sample T-test) that allows us to reject this hypothesis. As can be seen in Table 2, for MIMIC-III mortality prediction, we can reject the hypothesis that AUC difference is greater than 0.04 with *p*-value smaller than 0.01 (average AUC difference is 0.009). For eICU gender prediction, we achieve 0.019 average AUC difference with *p*-value smaller than 0.001.

### Privacy

**Fig. 2:**
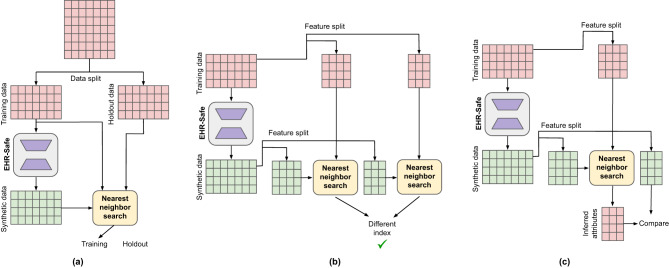
Block diagrams of three privacy metrics. Three privacy metrics used to evaluate the privacy risk of generated synthetic datasets. **a** Membership inference. **b** Re-identification. **c** Attribute inference.

Unlike de-identified data, there is no straightforward one-to-one mapping between real and synthetic data (generated from random vectors). However, there may be some indirect privacy leakage risks built on correlations between the synthetic data and partial information from real data. We consider three different privacy attacks that represent known approaches that adversaries may apply to de-anonymize private data (details are provided in Fig. 2 and Supplementary Information):

- **Membership inference attack**: The adversary explores the probability of data being a member of the training data used for training the synthetic data generation model^31^.
- **Re-identification attack**: The adversary explores the probability of some features being re-identified using synthetic data and matching to the training data^32^.
- **Attribute inference attack**: The adversary predicts the value of sensitive features using synthetic data^33^.

These metrics are highly practical as they represent the expected risks that currently prevent sharing of conventionally anonymized data. Furthermore, they are highly interpretable, as results for these metrics directly measure the risks associated with sharing synthetic data.

Table 3 summarizes the results along with the ideal achievable value for each metric. According to the results shown in Table 3, we observe that the privacy metrics are very close to the ideal in all cases. The risk of understanding whether a sample of the original data is a member used for training the model is very close to random chance. For the attribute inference attack, we focus on the prediction task of inferring specific attributes (gender, religion and marital status) using other attributes as features. We compare prediction accuracy when training a kNN classifier with real data against another kNN classifier trained with synthetic data. The results demonstrate that access to synthetic data does not lead to higher prediction performance on specific attributes as compared to access to the original data. More results for privacy with different distance metrics can be found in Supplementary Information.

**Table 3:** Privacy risk evaluation across three different metrics.

Privacy metrics		MIMIC-III		eICU	
		No privacy risk	EHR-Safe	No privacy risk	EHR-Safe
**Membership inference**		0.500	0.496	0.500	0.489
**Re-identification**		0.049	0.061	0.068	0.085
**Attribute inference**	Specific attributes	With original data	EHR-Safe	With original data	EHR-Safe
	Gender	0.696	0.681	0.678	0.669
	Marital status	0.628	0.620	-	-
	Religion	0.639	0.619	-	-

For membership inference, the ideal value is random guessing (i.e., 0.5) whether an original sample has been leveraged for training the synthetic data generation model. For the re-identification, the ideal case is to replace the synthetic data with holdout original data, which is disjoint with the training data. For attribute inference attack, we set three static features (gender, race, medical status—note that eICU only has a gender attribute) as the specific attributes and report the prediction AUC. The baseline scenario is measured by performing feature prediction using the original data. For multi-class data such as marital status or religion, we compute the pairwise AUCs across all possible categories and report their average values.

## Discussion

We provide ablation studies on key components of EHR-Safe in Table 4 (top): (1) stochastic normalization, (2) explicit mask modeling, and (3) categorical embedding. All three components are observed to substantially contribute to the quality of synthetic data generation. Supplementary Information further illustrates the impact of stochastic normalization in terms of CDF curves.

**Table 4:** Comparisons with alternatives.

Models		Fidelity				Privacy	
		MIMIC-III		eICU		MIMIC-III	eICU
		AUC	AP	AUC	AP	Membership Inference	
Upper bound—using real data		0.723	0.276	0.954	0.600	0.500	0.500
EHR-Safe		0.710	0.251	0.945	0.580	0.496	0.489
EHR-Safe variants	Without stochastic normalization	0.674	0.226	0.918	0.533	0.505	0.509
	Without explicit mask modeling	0.691	0.231	0.883	0.333	0.511	0.497
	Without categorical embedding	0.681	0.223	0.935	0.569	0.492	0.510
	TimeGAN	0.576	0.147	0.726	0.241	0.513	0.508
Alternatives	RC-GAN	0.554	0.129	0.684	0.245	0.506	0.514
	C-RNN-GAN	0.567	0.146	0.671	0.229	0.511	0.494

Downstream task performances (mortality prediction with RF model) and membership inference metrics (0.5 as the ideal case) with three different variations of EHR-Safe and three alternative models.

In Table 4 (bottom), we compare EHR-Safe to three alternative methods (TimeGAN^15^, RC-GAN^34^, C-RNN-GAN^35^) proposed for time-series synthetic data generation. Note that the alternative methods are not designed to handle all the challenges of EHR data, such as varying length sequences, missingness and joint representation of static and time-varying features (please see Supplementary Information on how we modify them for these functionalities). Thus, they significantly underperform EHR-Safe, as shown in Table 4.

Post-processing can further improve the statistical similarity of the synthetic data. Perfectly matching the distributions of synthetic and real data might be particularly challenging for features with skewness or CDFs with discrete jumps. For some scenarios where EHR-Safe might have a shortcoming in matching the distributions, a proposed post-processing method (details can be found in Supplementary Information) can further refine the generated data and improve the fidelity results for statistical similarity. The post-processing method is based on matching the ratios of samples in different buckets for the real and synthetic data. Note that this procedure is not a learning-based method (i.e., no trainable parameters). With this procedure, we can significantly improve the statistical similarity—KS-statistics are less or equal to 0.01 for all features. However, the drawbacks are the additional complexity of generating synthetic data and a slight degradation of the utility metrics (e.g., AUC changed from 0.749 to 0.730 on MIMIC-III with Random Forest). There is not much difference in the proposed privacy metrics (e.g., membership-inference attack metric changed from 0.493 to 0.489 on MIMIC-III).

We demonstrate that EHR-Safe achieves very strong empirical privacy results when considering multiple practical privacy metrics. However, EHR-Safe does not provide theoretical privacy guarantees (e.g., differential privacy) unless its training is modified by randomly perturbing the models^21,36^. Note that EHR-Safe framework can be directly adopted with differential privacy. For instance, DP-SGD^37^ can be used to train the encoder-decoder and WGAN-GP models to achieve a differentially private generator and decoder with respect to the original data. Since synthetic data are generated through the differentially private generator and decoder using the random vector as the inputs, the generated synthetic data are also differentially private with respect to the original data. Even though these approaches can be adopted to EHR-Safe, it may result in a decrease in fidelity as the added noise would hurt the generative model training.

For the proposed metrics, the specific assumptions and models might pose limitations. The proposed fidelity metrics that reflect the downstream machine learning use cases depend on the model type. For future work, it would be interesting to study which fidelity metrics would correspond to the performance of the best achievable model. Similarly, the proposed privacy attacks employ certain assumptions about the methodology and model of the attacker (e.g., nearest neighbor search for very high-dimensional data might be suboptimal). It would be interesting to understand the theoretically achievable privacy.

Most of our results are very close to the ideal achievable performance, indicating one could have high confidence in using our method in the real world. The result that has the most room for improvement is statistical similarity, as it is not as high for all features. Reducing this consistently across all features can be done with further advances in generative modeling.

Various follow-up directions remain important for future work. The EHR data of this paper’s focus are heterogeneous structured data, and we show significant advancement over the prior state-of-the-art that focused on more limited data types. A natural extension is to integrate the generative modeling capability for text and image data, as modern EHR datasets often contain both. Realistic generation of text and image data would require high capacity and deep decoders. However, such decoders would come with extra training challenges, and effective training of them could require a much higher number of data samples. In addition, extra training difficulties would arise due to the fact that training dynamics for different modalities are different. Utilizing *foundation models* that are pre-trained on publicly available data is shown to be one of the key drivers of the recent research progress for deep learning on image and text data (including generative modeling). However, publicly available general purpose image and text datasets often come from very different domains, and their relevance to real-world EHR data would be low.

In this paper, we verify the performance of EHR-Safe on two healthcare provider datasets which consist of admitted patients. An important follow-up work would be on applying EHR-Safe on out-patient medical datasets from primary care or insurance companies. Scaling synthetic data generation for a complete EHR dataset with many features is another important future work. From a modeling perspective, there is no fundamental limitation for scaling—EHR-Safe can be trained to generate a very high number of features without hitting computational issues. However, we expect degradation in the generation quality for rarely-observed features (e.g., almost 90% of the MIMIC-III features are measured less than 1 time per visit, on average). Weak data coverage would constitute the fundamental challenge.

In conclusion, we propose a generative modeling framework for EHR data, EHR-Safe, that can generate highly realistic synthetic EHR data that are robust to privacy attacks. EHR-Safe is based on generative adversarial networks modeling applied to the encoded representations of the raw data. We introduce multiple innovations in the EHR-Safe architecture and training mechanisms that are motivated by the key challenges in EHR data. These innovations enable EHR-Safe to demonstrate high fidelity (almost-identical properties with real data when desired downstream capabilities are considered) with almost-ideal privacy preservation.

## Methods

This research follows Google AI principles (https://ai.google/principles/), reviewed by Google Health Ethics Committee and solely publicly available datasets are used.

The overall EHR-Safe framework is illustrated in Fig. 1d. To synthesize EHR data, we adopt generative adversarial networks (GANs). EHR data are heterogeneous (see Fig. 1b), including time-varying and static features that are partially available. Direct modeling of raw EHR data is thus challenging for GANs. To circumvent this, we propose utilizing a sequential encoder-decoder architecture to learn the mapping from the raw EHR data to low-dimensional representations and vice versa.

While learning the mapping, esoteric distributions of various numerical and categorical features pose a great challenge; for example, some values or numerical ranges might be much more common, dominating the distribution, while the capability of modeling rare cases is crucial. Our proposed methods for feature mapping are key to handling such data by converting to distributions for which the training of encoder-decoder and GAN are more stable and accurate. The mapped low-dimensional representations, generated by the encoder, are used for GAN training, and at test time, they are generated, which are then converted to raw EHR data with the decoder. Algorithm 1 overviews the training procedure for EHR-Safe. In the following subsections, we explain the key components.

### Feature representations

EHR data often consist of both static and time-varying features. Each static and temporal feature can be further categorized into either numeric or categorical. Measurement time for time-varying features is another important feature. Overall, the five categories of features for the patient index *i* are: (1) measurement time as *u*, (2) static numeric feature (e.g., age) as **s**^*n*^, (3) static categorical feature (e.g., marital status) as **s**^*c*^, (4) time-varying numerical feature (e.g., vital signs) as **t**^*n*^, (5) time-varying categorical feature (e.g., hearth rhythm) as **t**^*c*^. The sequence length of time-varying features is denoted as *T*(*i*). Note that each patient record may have a different sequence length. With all these features, given training data can be represented as:

1 $${{{{D}}}}={\{{{{{\bf{s}}}}}^{n}(i),{{{{\bf{s}}}}}^{c}(i),{\{{u}_{\tau }(i),{{{{\bf{t}}}}}_{\tau }^{n}(i),{{{{\bf{t}}}}}_{\tau }^{c}(i)\}}_{\tau = 1}^{T(i)}\}}_{i = 1}^{N},$$

where *N* is the total number of patient records.

EHR datasets often contain missing features as patients might visit clinics sporadically, and not all measurements or information are collected completely at all visits. In order to generate realistic synthetic data, missingness patterns should also be generated in a realistic way. Let’s denote the binary mask *m* with 1/0 values based on whether a feature is observed (*m* = 1) or not (*m* = 0). The missingness for the features is represented as

2 $${{{{{D}}}}}_{{{{{M}}}}}={\{{{{{\bf{m}}}}}^{n}(i),{{{{\bf{m}}}}}^{c}(i),{\{{{{{\bf{m}}}}}_{\tau }^{n}(i),{{{{\bf{m}}}}}_{\tau }^{c}(i)\}}_{\tau = 1}^{T(i)}\}}_{i = 1}^{N}.$$

Note that there is no missingness for measurement time—we assume time is always given whenever at least one time-varying feature is observed.

**Fig. 3:**
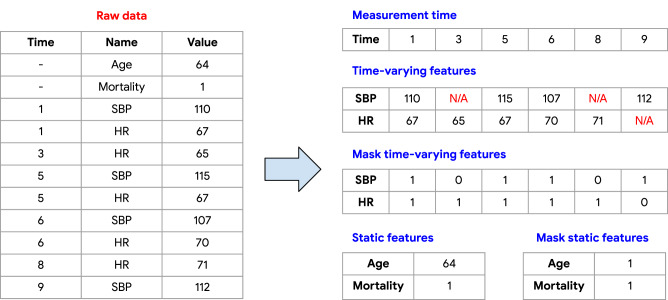
Converting raw data into multiple feature categories. Illustration of converting the raw data into multiple categories of features. The missing values of time-varying features are shown with N/A. Observed/missing values are represented with 1/0 in the mask features.

Figure 3 visualizes how the raw data are converted into four categories of features: (1) measurement time, (2) time-varying features, (3) mask features, (4) static features.

### Encoding and decoding categorical features

Handling categorical features poses a unique challenge beyond numerical features, as meaningful discrete mappings need to be learned. One-hot encoding is one possible solution; however, if some features have a large number of categories (such as the medical codes), the number of dimensions would significantly increase, hurting the GAN training and data efficiency^38^. We propose encoding and decoding categorical features to obtain learnable mappings to be used for generative modeling. We first encode the categorical features (**s**^*c*^) into one-hot encoded features (**s**^*co*^)—here, we use the notation with static categorical feature but it is the same with temporal categorical features. Then, we employ a categorical encoder (*CE*^*s*^) to transform one-hot encoded features into the latent representations (**s**^*ce*^):

3 $${{{{\bf{s}}}}}^{ce}=C{E}^{s}[{{{{\bf{s}}}}}^{co}]=CE[{s}_{1}^{co},...,{s}_{K}^{co}],$$

where *K* is the number of categorical features. Lastly, we use the multi-head decoders ($[C{F}_{1}^{s},...,C{F}_{K}^{s}]$) to recover the original one-hot encoded data from the latent representations.

4 $${\hat{{{{\bf{s}}}}}}_{k}^{co}=C{F}_{k}^{s}[{{{{\bf{s}}}}}^{ce}]$$

Both encoder (*CE*^*s*^) and multi-head decoders ($[C{F}_{1}^{s},...,C{F}_{K}^{s}]$) are trained with softmax cross entropy objective: (${{{{{L}}}}}_{c}$):

5 $$\mathop{\min }\limits_{C{E}^{s},C{F}_{1}^{s},...,C{F}_{K}^{s}}\mathop{\sum }\limits_{k=1}^{K}{{{{{L}}}}}_{c}(C{F}_{i}^{s}[CE[{s}_{1}^{co},...,{s}_{K}^{co}]],{s}_{i}^{co}).$$

We use separate encoder-decoder models for static and temporal categorical features. The transformed representations are denoted as **s**^*ce*^ and **t**^*ce*^, respectively.

#### Algorithm 1

Pseudo-code of EHR-Safe training.

**Input**: Original data ${{{{D}}}}={\{{{{{\bf{s}}}}}^{n}(i),{{{{\bf{s}}}}}^{c}(i),{\{{u}_{\tau }(i),{{{{\bf{t}}}}}_{\tau }^{n}(i),{{{{\bf{t}}}}}_{\tau }^{c}(i)\}}_{\tau = 1}^{T(i)}\}}_{i = 1}^{N}$

1: Generate missing patterns of ${{{{D}}}}$: ${{{{{D}}}}}_{{{{{M}}}}}={\{{{{{\bf{m}}}}}^{n}(i),{{{{\bf{m}}}}}^{c}(i),{\{{{{{\bf{m}}}}}_{\tau }^{n}(i),{{{{\bf{m}}}}}_{\tau }^{c}(i)\}}_{\tau = 1}^{T(i)}\}}_{i = 1}^{N}$

2: Transform categorical data (**s**^*c*^, **t**^*c*^) into one-hot encoded data (**s**^*co*^, **t**^*co*^)

3: Train static categorical encoder and decoder:

6 $$\mathop{\min }\limits_{C{E}^{s},C{F}_{1}^{s},...,C{F}_{K}^{s}}\mathop{\sum }\limits_{k=1}^{K}{{{{{L}}}}}_{c}(C{F}_{i}^{s}[CE[{s}_{1}^{co},...,{s}_{K}^{co}]],{s}_{i}^{co})$$

4: Train temporal categorical encoder and decoder:

7 $$\mathop{\min }\limits_{C{E}^{t},C{F}_{1}^{t},...,C{F}_{K}^{t}}\mathop{\sum }\limits_{k=1}^{K}{{{{{L}}}}}_{c}(C{F}_{i}^{t}[CE[{t}_{1}^{co},...,{t}_{K}^{co}]],{t}_{i}^{co})$$

5: Transform one-hot encoded data (**s**^*co*^, **t**^*co*^) to categorical embeddings (**s**^*ce*^, **t**^*ce*^)

6: Stochastic normalization for numerical features (**s**^*n*^, **t**^*n*^, *u*) (see Algorithm 2)

7: Train encoder-decoder model using Equation (11)

8: Generate original encoder states **e** using trained encoder (*E*), original data ${{{{D}}}}$ and missing patterns ${{{{{D}}}}}_{{{{{M}}}}}$

9: Train generator (*G*) and discriminator (*D*) using WGAN-GP

8 $$\mathop{\max }\limits_{G}\mathop{\min }\limits_{D}\frac{1}{N}\mathop{\sum }\limits_{i=1}^{N}D({{{\bf{e}}}}[i])-\frac{1}{N}\mathop{\sum }\limits_{i=1}^{N}D(\hat{{{{\bf{e}}}}}[i])+\eta [{(| | \nabla D(\tilde{{{{\bf{e}}}}}[i])| | -1)}^{2}]$$

**Output**: Trained generator (*G*), trained decoder (*F*), trained categorical decoder (*CF*^*s*^, *CF*^*t*^)

### Stochastic normalization for numerical features

One prominent challenge for training GAN is mode collapse^38^, i.e., the generative model overemphasizes the generation of some commonly observed data values. Especially for distributions where the mass probability is condensed within a small numerical range, this can be a severe issue. For EHR data, such distributions are indeed observed for many features.

Some numerical clinical features might have values from a discrete set of observations (e.g., high respiratory pressure values coming as multiples of 5—35, 40, 45, etc.) or from highly nonuniform distributions, yielding cumulative distribution functions (CDFs) that are discontinuous or with significant jumps.

Directly generating such numerical features coming from highly discontinuous CDFs can be challenging for GANs, as they are known to suffer from mode collapse and would have the tendency to generate common values for all samples. To circumvent this issue and obtain high fidelity, we propose a normalization/renormalization method, shown in Algorithms 2 and 3, that map the raw feature distributions to and from a more uniform distribution that is easier to model with GANs. An example application would be like: (1) estimate the ratio of each unique value in the original feature; (2) transform each unique value into the normalized feature space with the ratio as the width—if we have 3 original values: (1, 2, 3) and their corresponding ratios as (0.1, 0.7, 0.2); (3) map 1 into [0, 0.1] range in a uniformly random way; for 2, we map into [0.1, 0.8]; for 3, we map into [0.8, 1.0].

#### Algorithm 2

Pseudo-code of stochastic normalization.

**Input**: Original feature *X*

1: **Uniq(X)** = Unique values of *X*, **N** = Length of (*X*)

2: **lower-bound** = 0.0, **upper-bound** = 0.0, $\hat{X}=X$

3: **for** val in Uniq(X) **do**

4:   Find index of *X* whose value = val as **idx(val)**

5:   Compute the frequency (ratio) of val as **ratio(val)** = Length of idx(val) / N

6:   upper-bound = lower-bound + ratio(val)

7:   $\hat{X}$[idx(val)] ~ **Uniform**(lower-bound, upper-bound)

8:   params[val] = [lower-bound, upper-bound]

9:   lower-bound = upper-bound

10: **end** **for**

**Output**: Normalized feature ($\hat{X}$), normalization parameters (params)

#### Algorithm 3

Pseudo-code of stochastic renormalization.

**Input**: Normalized feature ($\hat{X}$), normalization parameters (params)

1: $$X=\hat{X}$$

2: **for** param in params.keys **do**

3:   Find index of $\hat{X}$ whose value is in [param.values] as **idx(param)**

4:   X[idx(param)] = param

5: **end** **for**

**Output**: Original feature *X*

As shown in Supplementary Information, the proposed stochastic normalization can be highly effective in transforming features with discontinuous CDFs into approximately uniform distributions while allowing for perfect renormalization into the original feature space. We demonstrate that the impact of normalization is significant for EHR-Safe to improve results in Table 4.

We also note that the stochastic normalization method is highly effective for handling skewed distributions that might correspond to features with outliers. Stochastic normalization maps the original feature space (with outliers) into a normalized feature space (with uniform distribution), and then the applied renormalization recreates the skewed distributions with outliers.

### Encoder-decoder architecture

Given the described encoding scheme for numerical and categorical features, next, we describe the employed architecture for jointly extracting the representations from multiple types of data, including static, temporal, measurement time, and mask features. We propose to encode these heterogeneous features into joint representations from which the synthetic data samples are generated. High-dimensional sparse data are challenging to model with GANs, as they might cause convergence stability and mode collapse issues, and they might be less data efficient^38^ To address this, using an encoder-decoder model is beneficial as it condenses high-dimensional heterogeneous features into latent representations that are low dimensional and compact.

**Fig. 4:**
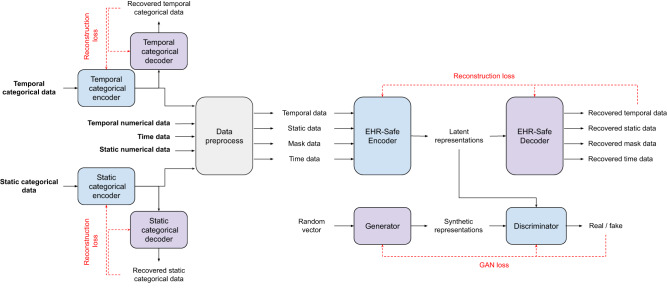
Block diagram of EHR-Safe training. Blue and purple blocks indicate trainable components, and gray blocks are non-trainable. Three pairs of encoder-decoder models are trained based on the reconstruction losses. The generator and discriminator models are trained by GAN loss.

The encoder model (*F*) inputs the static data (**s**^*n*^, **s**^*ce*^), temporal data (**t**^*n*^, **t**^*ce*^), time data (*u*), and mask data (${{{{\bf{m}}}}}^{n},{{{{\bf{m}}}}}^{c},{{{{\bf{m}}}}}_{\tau }^{n},{{{{\bf{m}}}}}_{\tau }^{c}$) and generates the encoder states (**e**), as shown in Fig. 4 and below equations.

9 $${{{\bf{e}}}}=E({{{{\bf{s}}}}}^{n},{{{{\bf{s}}}}}^{ce},{{{{\bf{t}}}}}^{n},{{{{\bf{t}}}}}^{ce},u,{{{{\bf{m}}}}}^{n},{{{{\bf{m}}}}}^{c},{{{{\bf{m}}}}}_{\tau }^{n},{{{{\bf{m}}}}}_{\tau }^{c})$$

The decoder model (*G*) inputs these encoded representations (**e**) and aims to recover the original static, temporal, measurement time, and mask data.

10 $${\hat{{{{\bf{s}}}}}}^{n},{\hat{{{{\bf{s}}}}}}^{ce},{\hat{{{{\bf{t}}}}}}^{n},{\hat{{{{\bf{t}}}}}}^{ce},\hat{u},{\hat{{{{\bf{m}}}}}}^{n},{\hat{{{{\bf{m}}}}}}^{c},{\hat{{{{\bf{m}}}}}}_{\tau }^{n},{\hat{{{{\bf{m}}}}}}_{\tau }^{c}=F({{{\bf{e}}}})$$

If the decoder model can recover the original heterogeneous data correctly, it can be inferred that **e** contains most of the information in the original heterogeneous data.

For temporal, measurement time and static features, we use mean square error (${{{{{L}}}}}_{m}$) as the reconstruction loss. Note that we compute the errors only when the features are observed. For the mask features, we use the binary cross entropy (${{{{{L}}}}}_{c}$) as the reconstruction loss because the mask features consist of binary variables. Thus, our full reconstruction loss becomes:

11 $$\begin{array}{ll}\min\, {{{{{L}}}}}_{c}({\hat{{{{\bf{m}}}}}}^{n},{{{{\bf{m}}}}}^{n})+{{{{{L}}}}}_{c}({\hat{{{{\bf{m}}}}}}^{c},{{{{\bf{m}}}}}^{c})+{{{{{L}}}}}_{c}({\hat{{{{\bf{m}}}}}}_{\tau }^{n},{{{{\bf{m}}}}}_{\tau }^{n})+{{{{{L}}}}}_{c}({\hat{{{{\bf{m}}}}}}_{\tau }^{c},{{{{\bf{m}}}}}_{\tau }^{c})+\\ \qquad\lambda [{{{{{L}}}}}_{m}(\hat{u},u)+{{{{{L}}}}}_{m}({{{{\bf{m}}}}}^{n}{\hat{{{{\bf{s}}}}}}^{n},{{{{\bf{m}}}}}^{n}{{{{\bf{s}}}}}^{n})+{{{{{L}}}}}_{m}({{{{\bf{m}}}}}^{c}{\hat{{{{\bf{s}}}}}}^{ce},{{{{\bf{m}}}}}^{c}{{{{\bf{s}}}}}^{ce})+{{{{{L}}}}}_{m}({{{{\bf{m}}}}}_{\tau }^{n}{\hat{{{{\bf{t}}}}}}^{n},{{{{\bf{m}}}}}_{\tau }^{n}{{{{\bf{t}}}}}^{n})+{{{{{L}}}}}_{m}({{{{\bf{m}}}}}_{\tau }^{c}{\hat{{{{\bf{t}}}}}}^{ce},{{{{\bf{m}}}}}_{\tau }^{c}{{{{\bf{t}}}}}^{ce})],\end{array}$$

where *λ* is the hyper-parameter to balance the cross entropy loss and mean squared loss.

### Adversarial training

The trained encoder model is used to map raw data into encoded representations, that are then used for GAN training so that the trained generative model can generate realistic encoded representations that can be decoded into realistic raw data.

We first utilize the trained encoder to generate original encoder states (*e*) using the original raw data—the original dataset gets converted into ${{{{{D}}}}}_{e}={\{{{{\bf{e}}}}(i)\}}_{i = 1}^{N}$. Next, we use the generative adversarial network (GAN) training framework to generate synthetic encoder states $\hat{{{{\bf{e}}}}}$ to make synthetic encoder states dataset ${\hat{{{{{D}}}}}}_{e}$. More specifically, the generator (*G*) uses the random vector (**z**) to generate synthetic encoder states as follows.

12 $$\hat{{{{\bf{e}}}}}=G({{{\bf{z}}}})$$

Then, the discriminator *D* tries to distinguish the original encoder states **e** from the synthetic encoder states $\hat{{{{\bf{e}}}}}$. As the GAN framework, we adopt Wasserstein GAN^39^ with Gradient Penalty^40^ due to its training stability for heterogeneous data types. The optimization problem can be stated as:

13 $$\begin{array}{ll}\mathop{\max }\limits_{G}\mathop{\min }\limits_{D}\frac{1}{N}\mathop{\sum }\limits_{i=1}^{N}D({{{\bf{e}}}}[i])-\frac{1}{N}\mathop{\sum }\limits_{i=1}^{N}D(\hat{{{{\bf{e}}}}}[i])+\eta [{(| | \nabla D(\tilde{{{{\bf{e}}}}}[i])| | -1)}^{2}]\\ \,{{\mbox{where}}}\,\,\tilde{{{{\bf{e}}}}}[i]=\epsilon {{{\bf{e}}}}[i]+(1-\epsilon )\hat{{{{\bf{e}}}}}[i]\,{{\mbox{and}}}\,\epsilon \sim U[0,1],\end{array}$$

where *η* is WGAN-GP hyper-parameter, which is set to 10. Figure 4 describes the proposed GAN model with generator and discriminator architectures based on multi-layer perceptron (MLP).

### Inference

The inference process of EHR-Safe is overviewed in Algorithm 4. After training both the encoder-decoder and GAN models, we can generate synthetic heterogeneous data from any random vector. Note that only the trained generator and decoder are used for inference.

**Fig. 5:**
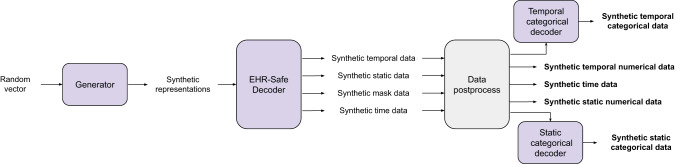
Block diagram of EHR-Safe model inference. Generator converts random vectors into synthetic representations. Then, decoders convert the syntheticrepresentations to synthetic temporal/static/time data.

As shown in Fig. 5, the trained generator uses the random vector to generate synthetic encoder states.

14 $$\hat{{{{\bf{e}}}}}=G({{{\bf{z}}}})\,{{\mbox{where}}}\,{{{\bf{z}}}} \sim {{{{N}}}}(0,I)$$

Then, the trained decoder (*F*) uses the synthetic encoder states as the inputs to generate synthetic temporal (${\hat{{{{\bf{t}}}}}}^{n},{\hat{{{{\bf{t}}}}}}^{ce}$), static (${\hat{{{{\bf{s}}}}}}^{n},{\hat{{{{\bf{s}}}}}}^{ce}$), time ($\hat{u}$), and mask (${\hat{{{{\bf{m}}}}}}^{n},{\hat{{{{\bf{m}}}}}}^{c},{\hat{{{{\bf{m}}}}}}_{\tau }^{n},{\hat{{{{\bf{m}}}}}}_{\tau }^{c}$) data.

15 $${\hat{{{{\bf{s}}}}}}^{n},{\hat{{{{\bf{s}}}}}}^{ce},{\hat{{{{\bf{t}}}}}}^{n},{\hat{{{{\bf{t}}}}}}^{ce},\hat{u},{\hat{{{{\bf{m}}}}}}^{n},{\hat{{{{\bf{m}}}}}}^{c},{\hat{{{{\bf{m}}}}}}_{\tau }^{n},{\hat{{{{\bf{m}}}}}}_{\tau }^{c}=F(\hat{{{{\bf{e}}}}})$$

**Fig. 6:**
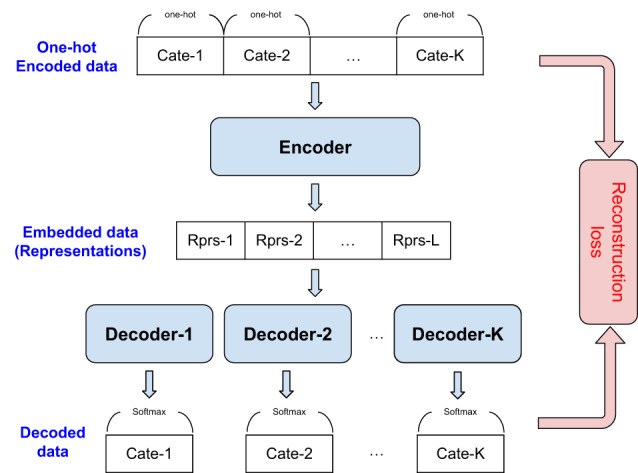
Encoder-decoder for categorical features. Encoder-decoder architecture to convert the categorical features into latent representations. Here, we use the multi-layer perceptron as the base model of the encoder-decoder architecture.

Representations for the static and temporal categorical features are decoded using the decoders in Fig. 6 to generate synthetic static categorical (${\hat{s{{{\boldsymbol{}}}}}}^{c}$) data and temporal categorical (${\hat{{{{\bf{t}}}}}}^{c}$) data.

16 $${\hat{{{{\bf{s}}}}}}^{c}=C{F}^{s}({\hat{{{{\bf{s}}}}}}^{ce}),{\hat{{{{\bf{t}}}}}}^{c}=C{F}^{t}({\hat{{{{\bf{t}}}}}}^{ce})$$

The generated synthetic data are represented as:

17 $$\hat{{{{{D}}}}}={\{{\hat{{{{\bf{s}}}}}}^{n}(i),{\hat{{{{\bf{s}}}}}}^{c}(i),{\{{\hat{{{{\bf{u}}}}}}_{\tau }(i),{\hat{{{{\bf{t}}}}}}_{\tau }^{n}(i),{\hat{{{{\bf{t}}}}}}_{\tau }^{c}(i)\}}_{\tau = 1}^{\hat{T}(i)}\}}_{i = 1}^{M}$$

18 $${\hat{{{{{D}}}}}}_{{{{{M}}}}}={\{{\hat{{{{\bf{m}}}}}}^{n}(i),{\hat{{{{\bf{m}}}}}}^{c}(i),{\{{\hat{{{{\bf{m}}}}}}_{\tau }^{n}(i),{\hat{{{{\bf{m}}}}}}_{\tau }^{c}(i)\}}_{\tau = 1}^{\hat{T}(i)}\}}_{i = 1}^{M}$$

Note that with the trained models, we can generate an arbitrary number of synthetic data samples (even more than the original data).

#### Algorithm 4

Pseudo-code of EHR-Safe inference.

**Input**: Trained generator (*G*), trained decoder (*F*), the number of synthetic data (*M*), trained categorical decoder (*CF*^*s*^, *CF*^*t*^)

1: Sample *M* random vectors ${{{\bf{z}}}} \sim {{{{N}}}}(0,I)$

2: Generate synthetic embeddings: $\hat{{{{\bf{e}}}}}=G({{{\bf{z}}}})$

3: Decode synthetic embeddings to synthetic data: ${\hat{{{{\bf{s}}}}}}^{n},{\hat{{{{\bf{s}}}}}}^{ce},{\hat{{{{\bf{t}}}}}}^{n},{\hat{{{{\bf{t}}}}}}^{ce},\hat{u},{\hat{{{{\bf{m}}}}}}^{n},{\hat{{{{\bf{m}}}}}}^{c},{\hat{{{{\bf{m}}}}}}_{\tau }^{n},{\hat{{{{\bf{m}}}}}}_{\tau }^{c}=F(\hat{{{{\bf{e}}}}})$

4: Decode synthetic categorical embeddings: ${\hat{{{{\bf{s}}}}}}^{c}=C{F}^{s}({\hat{{{{\bf{s}}}}}}^{ce}),{\hat{{{{\bf{t}}}}}}^{c}=C{F}^{t}({\hat{{{{\bf{t}}}}}}^{ce})$

5: Renormalize synthetic numerical data (${\hat{{{{\bf{s}}}}}}^{n},{\hat{{{{\bf{t}}}}}}^{n},\hat{u}$) (see Algorithm 3)

**Output**: Synthetic data $\hat{{{{{D}}}}}={\{{\hat{{{{\bf{s}}}}}}^{n}(i),{\hat{{{{\bf{s}}}}}}^{c}(i),{\{{\hat{{{{\bf{u}}}}}}_{\tau }(i),{\hat{{{{\bf{t}}}}}}_{\tau }^{n}(i),{\hat{{{{\bf{t}}}}}}_{\tau }^{c}(i)\}}_{\tau = 1}^{\hat{T}(i)}\}}_{i = 1}^{M}$ and synthetic missing pattern ${\hat{{{{{D}}}}}}_{{{{{M}}}}}={\{{\hat{{{{\bf{m}}}}}}^{n}(i),{\hat{{{{\bf{m}}}}}}^{c}(i),{\{{\hat{{{{\bf{m}}}}}}_{\tau }^{n}(i),{\hat{{{{\bf{m}}}}}}_{\tau }^{c}(i)\}}_{\tau = 1}^{\hat{T}(i)}\}}_{i = 1}^{M}$

### Supplementary information

Supplementary Information

## Data Availability

The data used for the training, validation, and test sets are publicly available. All data were collected entirely from openly available sources. The following websites can be used to access the EHR datasets used in this study: MIMIC-III (https://physionet.org/content/mimiciii/1.4/), eICU (https://eicu-crd.mit.edu/gettingstarted/access/).
