# Supplementary material for: EHR-Safe: generating high-fidelity and privacy-preserving synthetic electronic health records
Source: NPJ Digit Med. 2023 Aug 11;6:141. doi: 10.1038/s41746-023-00888-7 (PMC10421926; doi:10.1038/s41746-023-00888-7)
Supplement: Supplementary file 1 — Supplementary Information [file 41746_2023_888_MOESM1_ESM.pdf]

Supplementary Information:

EHR-Safe: Generating High-Fidelity and Privacy-Preserving Synthetic Electronic Health Records

Jinsung Yoon*, Michel Mizrahi, Nahid Farhady Ghalaty, Thomas Jarvinen, Ashwin S. Ravi, Peter Brune, Fanyu Kong, Dave Anderson, George Lee, Arie Meir, Farhana Bandukwala, Elli Kanal, Sercan Ö. Arık, Tomas Pfister

Corresponding author: Jinsung Yoon (jinsungyoon@google.com)*

Google Cloud

1 Supplementary Methods: Evaluation framework details

1.1 Fidelity metrics

For generative modeling, there is no standard way of evaluating the fidelity of the generated synthetic data samples and often different works based their evaluations on different methods. Therefore, we use various methods to provide the fidelity results including (i) training on synthetic / testing on real, (ii) KS-statistics, (iii) CDF graphs, (iv) feature importance.

Regarding the rationale for (i), the common use-case of synthetic data is to construct ML models for downstream tasks. To construct downstream ML models without utilizing the original data, we envision the scenario of first generating synthetic data and then sharing the synthetic data to ML developers, will be quite common. Then, in the ideal case, ML developers would be able to construct downstream models using the synthetic data that can match the performance as if they were trained on real data. Therefore, we use (i) as our primary metric for quantifying whether our synthetic data can provide satisfactory performance on downstream learning tasks. Note that metric (iv) is inherently related to (i) as failure to capture the properties of important features in the real data would be expected to hurt performance of downstream models. Metric (ii) is adopted as the fundamental statistical fidelity metric for our synthetic data, as it is both interpretable, and sufficiently general to handle the diverse data modalities addressed in our work.

KS-statistics can be computed for binary, categorical, and continuous data; and in all cases, results can be interpreted as the maximum distribution distance in terms of probability.

1.1.1 Distribution distance

We employ KS-statistics to quantify the distances between the distributions of real and synthesized samples. KS-statistics is a non-parametric test that can be used to compare two samples, and provide a probability that both collections were drawn from the same probability distribution. It quantifies the distance between the empirical distribution functions of the two samples. If the computed KS-statistic value is small (or the corresponding p-value (computed by Kolmogorov-Smirnov test) is high), we can say that the null hypothesis can be rejected. The null hypothesis in this case states that the distribution of the original and synthetic data (for one specific feature) is the same.

1.1.2 Utility metric

The utility metric focuses on usefulness of the synthetic data for a given task. One common use case of synthetic data would be developing predictive models on them without access to the real data. The ideal scenario would be synthetic and real data having sufficiently similar characteristics that they would yield similar models (when the same model development procedure is applied on them), and eventually similar predictions on the unseen real data (please see [1] for a more comprehensive discussion). It should be noted that choice of training data, features to predict, and models to train can all have a big impact on observed utility. For instance, low capacity or poorly-tuned models may result in low accuracy regardless of the quality of the synthetic data. Throughout this paper, we present numerous results of the downstream model performance that shows that the real data can be replaced with synthetic data with minimal performance penalty.

1.1.3 Multi-target utility metric

The utility metric is inherently limited as a measure of the realisticness of synthetic data, as in the general case, similarity of model performance does not strictly imply similarity of the underlying training data. For instance the target feature may be completely unrelated with the rest of the dataset, leading to equally bad performance regardless of the training data being used. Instead, the utility metric attempts to measure the preservation of predicatively useful statistical properties of the underlying data. However, only measuring utility with respect to a single target variable, may fail to capture important aspects of the data. Additionally, only considering the scenario where all features are available for prediction may fail to capture the utility of features of lesser importance for the predictive task under consideration.

To address these limitations, we propose a framework to more comprehensively evaluate utility. In order to validate that all features are well-preserved in the synthetic data, we can compute utility using every possible subset of features as predictors (instead of using all features). If for each possible subset of features, a common model trained on both datasets always makes similar predictions, then we can make stronger claims about the usefulness of the synthetic dataset. To further validate the utility metric under diverse settings, we can measure performance when predicting any feature (which is not part of the training data) instead of fixing on only one such as mortality. In this paper, we focus on predicting only static-categorical data.

There are 2^n subsets of features, which makes the task of running the utility-metric using each possible subset a computationally unfeasible task. Instead, we can use the hypothesis-testing framework and work with a confidence level. This approach will make our approach computationally feasible while giving us a confidence level on our results.

We state our null hypothesis (H0) as follows: The mean of the absolute difference between the models trained on real and synthetic data measured using metric **M** on predicting any categorical static feature **F** with model **P** using any set of features is greater or equal than **X**.

We use Random Forest (RF) **P** due to its high accuracy while being relatively fast for training. We randomly choose in each experiment the target feature to predict **F** among the available categorical static variables (mortality, gender, condition-code, marital-status and religion). We also choose a random subset of features to use for prediction, while avoiding to use the feature **F**. We run the experiment $n = 30$ times and compute the Area Under the Receiver Operating Characteristic Curve (AUC) as our metric **M**. Then, we compute the statistical test that the mean of the underlying distribution of the sample (i.e. the n results representing the absolute differences between training-real and training-synthetic) is greater than the given population mean (i.e. the percent we are using in our hypothesis or $\frac{X}{100}$). We did this only for MIMIC dataset as for the eICU dataset only two target variables were available (mortality and gender), both of which we used for the results in the main manuscript. The mean of the differences was 0.057. For $X = 6$, the p-value (computed by one sample T-test) to reject the hypothesis was 0.052.

1.2 Privacy metrics

In this section, we overview the privacy attacks against the model. We choose three privacy metrics that represent known approaches which adversaries may apply to de-anonymize private data – (i) membership inference, (ii) re-identification, (iii) attribute inference. These metrics are highly practical as they represent the expected risks that currently prevent sharing of conventionally anonymized data. Furthermore, they are

highly interpretable, as results for these metrics directly measure the risks associated with sharing synthetic data. For instance, membership inference is a common attack for extracting protected attributes from private data. The ability for attackers to identify patients within a dataset also gives access to any sensitive attributes within the data. Thus, demonstrating resilience to membership inference attacks is a necessary condition for creating synthetic medical records that are safe to share. Furthermore, this metric can evaluate whether the generative model just memorizes the training data or learns the distributions of the original data which is critical for privacy. For (ii) and (iii) privacy metrics are about whether we can identify the private attributes if some non-private attributes are revealed. These are also highly practical and easy to interpret privacy metrics.

Our assumption for these attacks is that the adversary can be either in possession of the original data or the synthetically generated data. The attacks below cover the scenarios where the adversary only has access to the data rather than the model and does not cover the insider threat cases where the model is in the hands of the malicious party.

1.2.1 Membership inference attack

The adversary's goal with this attack is to understand whether an individual's data has been used for training the synthetic data generation model. In this case, the specific attribute is whether an individual has participated in a medical study. To evaluate the risk of this attack, we first divide the original data into training (50%) and holdout data (50%), and train EHR-Safe using only the training split. After generating the synthetic data, we train a k nearest neighbor (kNN) model fitted on the synthetic data. We assume that the adversary is in possession of real data containing both training data and holdout samples. Using the kNN model, we identify the closest neighbors for each sample in the real dataset. Using a minimal threshold line (e.g. minimum Hamming Distance), we predict whether the real data sample belongs to the training data. Then, we calculate the accuracy, since we are in possession of the labels (unlike in the case of a real attacker). For an ideal model (without privacy risk), the prediction accuracy would be 50%. If EHR-Safe had high privacy leakage, the kNN model would lead to higher accuracy.

1.2.2 Re-identification attack

The re-identification attack is a linkage attack analysing if a certain subset of features suggests that the synthesized sample belongs to a certain individual, then the same suggestion also holds with another subset of features for the same sample. We define the re-identification ratio utilizing the feature subset proximity, as a way of robustness against this linkage attack. We first divide the synthetically generated dataset into two

subsets based on the features. We simply use half of the features in each subset. Then, we find the nearest neighbors of each sub dataset from the original dataset to find one-to-one mapping between synthetic data and original data. Finally, we check whether these one-to-one mappings are consistent between two subsets. If they are consistent, we treat that sample as re-identifiable. The optimal value with this metric (i.e. no privacy risk) can be computed by replacing the synthetic data into disjoint holdout original data.

1.2.3 Attribute inference attack

For this attack, the adversary has some partial information about some individuals and based on correlating that information with the synthetic data, the attack analyses their ability to infer the specific attributes more accurately. We focus on gender, age, and race as the sensitive features for the experiments. Then, using the rest of the features, we assess the predictability of the values of these sensitive features. As the baseline, we consider predicting these sensitive features using original data.

2 Supplementary Methods: Post-processing to minimize distribution distance

When the fidelity metric of KS-statistics optimization is considered, further improvements can be obtained with a post-processing procedure to refine the distributions. We propose a post-processing method that is applied to each feature individually to optimize the resulting KS-statistic between each pair of features. In order to understand the method for optimizing, we start by giving a brief overview of the KS-statistic computation:

- Samples from both datasets are concatenated and sorted;
- For each observation, estimate the CDF of original and synthetic data, as well as their absolute difference;
- Compute the survival function over the maximum difference.

The goal of the proposed post-processing method is to define an arbitrary value $dist_{max}$ as the maximum acceptable CDF difference between synthetic and real data, and find a set of minimal changes to the synthetic dataset such that the $dist_{max}$ criteria is satisfied. Define S_o as the samples from the original dataset, and S_s as the samples from the synthetic dataset, both in non-decreasing order. Define $\text{count}(S, x) : |y \in S | y \leq x|$ (Amount of samples from S less or equal than x) and $\text{CDF}(S, x) : \frac{\text{count}(S,x)}{|S|}$

Recall the optimization goal is $|\text{CDF}(S_0, x) - \text{CDF}(S_s, x)| \leq dist_{max}$ and only values from S_s can be modified to satisfy the condition. Thus, if there exists an x such that $|\text{CDF}(S_0, x) - \text{CDF}(S_s, x)| > dist_{max}$, we apply

the optimization procedure. Let's rewrite the original equation first, which represents the final state for each x after the optimization:

1. $\text{count}(S_s, x) \leq (\frac{\text{count}(S_o,x)}{|S_o|} + dist_{max}) \times |S_s|$
2. $\text{count}(S_s, x) \geq (\frac{\text{count}(S_o,x)}{|S_o|} - dist_{max}) \times |S_s|$.

We want to minimize the amount of modifications to S_s while satisfying these inequalities. Thus, we only need to modify elements until the elements are equal, more modifications may also satisfy the inequality but will require more changes:

1. $\text{count}(S_s, x) = (\frac{\text{count}(S_o,x)}{|S_o|} + dist_{max}) \times |S_s|$
2. $\text{count}(S_s, x) = (\frac{\text{count}(S_o,x)}{|S_o|} - dist_{max}) \times |S_s|$.

Let's define $N_+ = (\frac{\text{count}(S_o,x)}{|S_o|} + dist_{max}) \times |S_s|$ and $N_- = (\frac{\text{count}(S_o,x)}{|S_o|} - dist_{max}) \times |S_s|$. Given $dist_{max} \geq 0$ and $\frac{\text{count}(S_o,x)}{|S_o|} \leq 1$ we can affirm that $N_- \leq |S_s|$, thus we set $N = N_-$. Note that $dist_{max}$ will be close to zero, therefore in most cases $N_+ \leq |S_s|$. In the cases where $N_+ > |S_s|$, set $N = |S_s|$, otherwise set $N = N_+$. Therefore, we need to modify S_s such that $\text{count}(S_s, x) = N$. There are many possible ways to modify S_s in order to have the first N values less or equal to x, and the rest of the values greater than x. Below is the approach we propose:

1. For the first N values of S_s: Change any value greater than x with x
2. For the rest of the $|S_s| - N$ values, replace every value that is less or equal to x with value y where y is the smallest value that belongs to S_s and is greater than x.

Note that we always use the replacement values existing in either S_s or S_o. This avoids adding values that are nonexistent in the given sets. We use $dist_{max} = 0$ for all the features. As Fig. 1 shows, the KS-statistics can drastically improve with the proposed procedure.

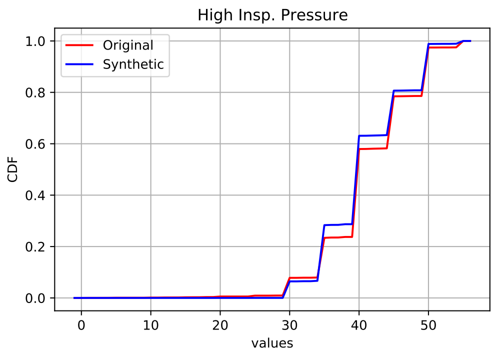

(a) KS-value: 0.05 p-value: 0.0

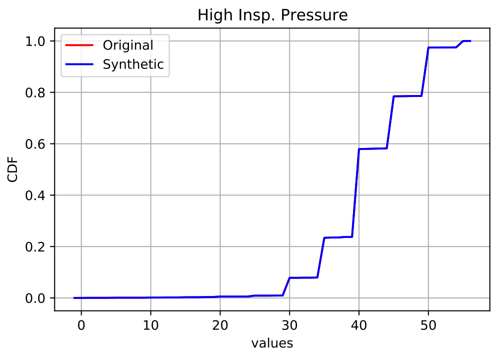

(b) KS-value: 0.00 p-value: 1.0

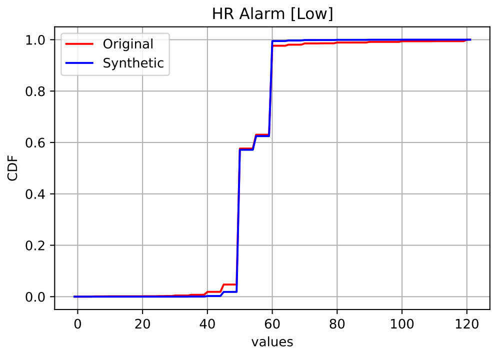

(c) KS-value: 0.03 p-value: 0.0

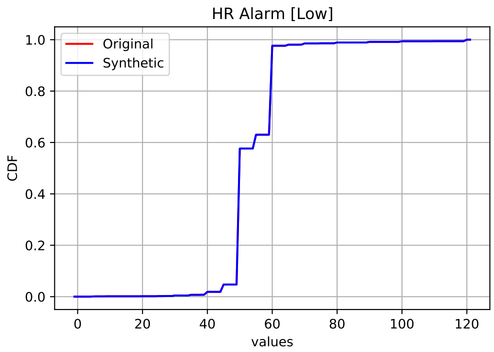

(d) KS-value: 0.00 p-value: 1.0

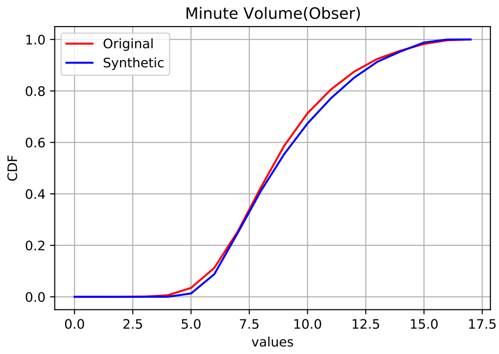

(e) KS-value: 0.04 p-value: 0.0

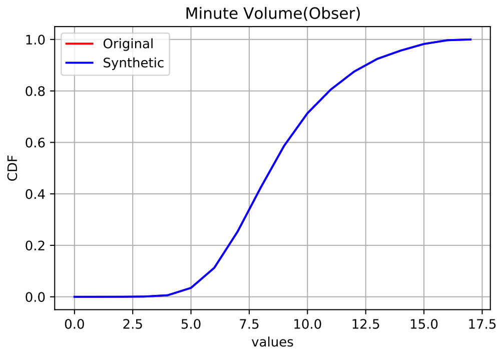

(f) KS-value: 0.00 p-value: 1.0

Figure 1: **CDF curves between original and synthetic data before (left) and after (right) post-processing** Here, we use 3 features as examples. (a,b) High Insp. Pressure, (c,d) HR Alarm[Low], (e,f) Minute Volume (Observed). P-values are computed by Kolmogorov-Smirnov test.

3 Supplementary Methods: Listwise feature generation

Listwise feature is another common data type in EHR data that represents a list of components at single measurement time. For instance, ICD-9 or ICD-10 codes at certain time point for a specific patient can be multiple; those are represented as listwise features. More examples can be found in Fig. 2.

Static listwise feature	
**Patient id**	**Condition code**
1	276.6, E87.70
2	573.9, K76.9
3	276.1, E87.0, E87.1
4	323.8, M32.19, G05.3
5	807.4
6	995.92, R65.2
7	294.10, 331.0, F02.8, G30.9

Temporal listwise feature		
**Patient id**	**Measurement time**	**Diagnosis code**
1	1	428.0, I50.9, 585.9, N18.9
1	2	414.00, I25.10, 491.20, J44.9
1	4	427.31, I48.0
2	1	286.9, D68.9, 345.90, R56.9
2	3	294.9, F03
3	1	428.1, I50.1, 584.9, N17.9
3	5	530.82, K22.8

Figure 2: **Examples of listwise features in EHR data** (left) static, (right) temporal.

Generating synthetic listwise features would be important to generate realistic healthcare synthetic data. Fortunately, with small modifications, EHR-Safe can generate synthetic listwise features. We describe the details of the modified EHR-Safe framework to generate listwise features in the following sections.

3.1 Data preprocessing

The number of values in each listwise feature per patient can be different. We first aggregate those multiple components and convert them into a numerical (binary) matrix.

id	Time	Temporal listwise feature
1	1	A, E
1	2	B, C
2	1	C, E
2	2	A, D, F
2	5	D, E, F
3	1	A, B
3	4	C

id	Time	Temporal listwise feature					
		A	B	C	D	E	F
1	1	1	0	0	0	1	0
1	2	0	1	1	0	0	0
2	1	0	0	1	0	1	0
2	2	1	0	0	1	0	1
2	5	0	0	0	1	1	1
3	1	1	1	0	0	0	0
3	4	0	0	1	0	0	0

Figure 3: **Listwise feature preprocessing** Converting listwise features into numeric matrix.

As can be seen in Fig. 3, the number of columns is the number of unique component in listwise features. Each row represents the unique patient id and measurement time. Present/absence of the component is

represented as 1/0 in the converted numerical matrix. This is very similar with categorical data preprocessing except the number of 1s in each row; listwise features can have multiple 1s in each row.

3.2 Encoder-decoder framework with listwise features

After encoding the listwise features, we can incorporate those encoded features into the categorical encoder and decoder framework.

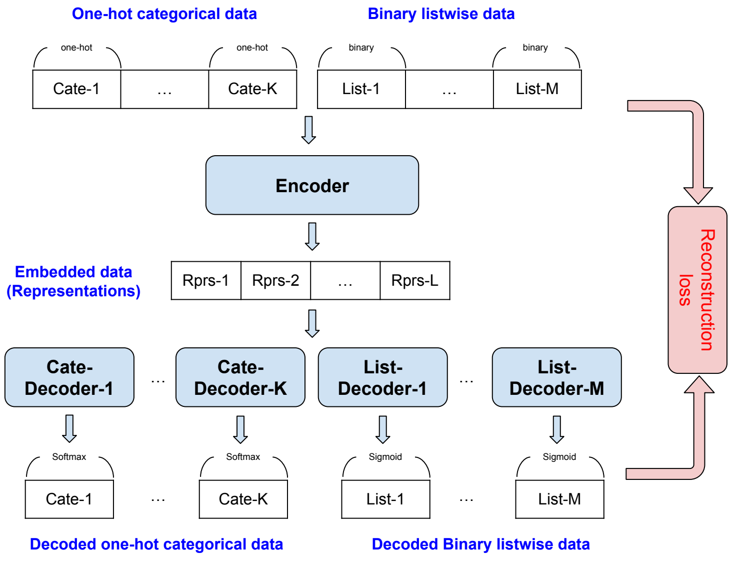

Figure 4: **Encoder-decoder for listwise features** Encoder-decoder architecture to convert both categorical and listwise features into the latent representations. Note that the listwise feature decoder uses sigmoid as the activation function.

As shown in Fig. 4, the overall architecture is highly overlapped with categorical encoder-decoder framework. One small difference is that we use sigmoid output activation function for the listwise decoder (instead of softmax) because multiple 1s can be possible in the converted listwise features in each row. The embedded representations include both categorical and listwise feature information.

3.3 Fidelity of the synthetic listwise features

To evaluate the fidelity of the synthetic listwise features, we first illustrate some example synthetic listwise features for some patients. As can be seen in Fig. 5, the generated synthetic listwise features are diverse and realistic. One interesting and important point is that we generate ICD-9 and ICD-10 codes independently but those are exactly matched in the synthetic listwise features.

We also plot the frequency of the listwise features in Fig. 6 which shows that the frequencies of top 10 features in both condition code and diagnosis are well aligned.

Synthetic static and temporal listwise features	
**Condition code**	**Diagnosis**
Other categories	038.9,A41.9,584.9,N17.9,R78.81,O
Other categories	038.9,A41.9,Other categories
414.00,I25.10	414.00,I25.10,401.9,I10
518.82	427.31,I48.0,780.09,584.9,N17.9,
518.82	427.31,I48.0,E87.1,348.30,G93.40
276.7,E87.5	491.20,J44.9,584.9,N17.9,799.02,
578.9,K92.2	518.82,038.9,A41.9,486,J18.9,585
Other categories	518.82,428.1,I50.1,584.9,N17.9,7
Other categories	585.9,N18.9,345.90,401.9,I10
Other categories	780.09,E980.2,Other categories

Original static and temporal listwise features	
**Condition code**	**Diagnosis**
414.00, I25.10	414.00,I25.10,584.9,N17.9
518.81,J96.00	427.31,I48.0,518.81,J96.00,427.5
414.00,I25.10,038.9,A41.9,428.1	518.81,J96.00,436,I67.8
414.00,I25.10,038.9,A41.9,428.1	491.20,J44.9,401.9,I10
Other categories	427.31,I48.0,Other categories
Other categories	427.31,I48.0,486,J18.9,348.30,G9
Other categories	427.31,I48.0,486,J18.9,348.30,G9
786.50,R07.9	410.71,I21.4,786.50,R07.9
Other categories	Other categories
340,G35	585.9,N18.9

Figure 5: **Examples of original and generated listwise features** (left) Generated synthetic listwise features and (right) original listwise feature.

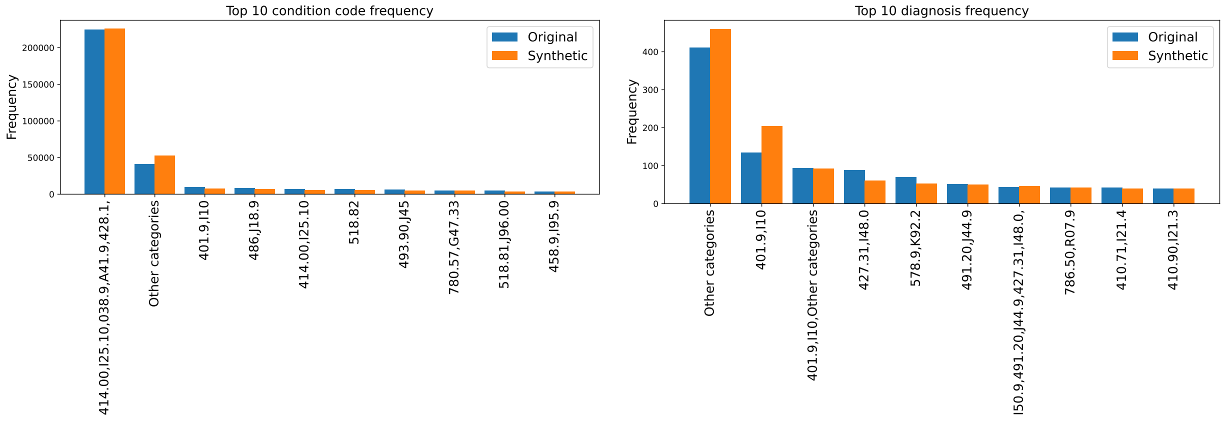

(a) Frequencies of condition codes

(b) Frequencies of diagnosis

Figure 6: **Top 10 components in both static and temporal listwise features** The frequencies between original and synthetic listwise features are well aligned.

4 Supplementary Methods: Training details and hyperparameters

In this section, we describe the details of EHR-Safe model training and hyper-parameters that we used. First, we divide the entire dataset into disjoint train (80%) and test (20%). Note that we only use the training data to train EHR-Safe model. Then, we preprocess the original data including normalization and padding. Note that to guarantee monotonicity of the measurement time, we model the time difference instead of the absolute time (which also included in the preprocessing). Then, we start EHR-Safe model training sequentially as follows: (i) train categorical (including temporal) embedders to convert categorical data into the categorical embedding, (ii) train encoder-decoder model, (iii) train WGAN-GP model. We summarize the values of the important hyperparameters used in EHR-Safe in Table 1.

**Hyper-parameters**	**MIMIC-III**	**eICU**
Static categorical embedder dimensions	30	30
Static categorical embedder epochs	100	100
Temporal categorical embedder dimensions	30	-
Temporal categorical embedder epochs	30	-
Encoder-decoder hidden dimensions	1000	1000
Encoder-decoder epochs	1500	1000
Batch size	256	256
WGAN-GP epochs	600	500
WGAN-GP gradient penalty weight	10.0	10.0
The number of WGAN-GP generator layers	4	4
The number of WGAN-GP discriminator layers	2	2
WGAN-GP hidden dimensions	2000	2000
Loss weights (temporal, mask, static, time)	(1.0, 1.0, 1.0, 0.1)	(1.0, 1.0, 1.0, 0.1)

Table 1: Hyperparameters to train EHR-Safe model for MIMIC-III and eICU datasets.

4.1 Alternative model training

In the main manuscript, we evaluate the utility and privacy performances of generated synthetic data by alternatives: (i) TimeGAN [2] (`https://github.com/jsyoon0823/TimeGAN`), (ii) RC-GAN [1] (`https://github.com/ratschlab/RGAN`), (iii) C-RNN-GAN [3] (`https://github.com/olofmogren/c-rnn-gan`). Note that the alternatives are not designed to handle various challenges of EHR data including varying lengths of sequences, sparsity, categorical features, and joint representation of static and time-varying features. To address those challenges with alternative methods, we introduce the following modifications.

- Varying lengths of sequences: Use padding approaches to make fixed length of sequences
- Sparsity: Use missing indicator to identify the missing components
- Categorical features: Use the integer encoding to convert string categories to integer. We avoid using one-hot encoding due to the large number of categories per each categorical feature.
- Joint representation of static and time-varying features: We treat the static features as duplicated time-series features for joint modeling.

5 Supplementary Note: Dataset details

Table 2 summarizes the key properties of the two EHR datasets used in our experiments. For MIMIC-III data, instead of including all the temporal numerical features, we only include top 75 features with high information gain which is computed by the Kullback-Liebler (KL) and Jensen-Shannon (JS) divergence between positive and negative labeled samples.

**Datasets**	**MIMIC-III**	**eICU**
Number of patients	19,946	198,707
Length of sequences (25% - 50% - 75% percentiles)	20 - 23 - 24	22 - 42 - 77
Maximum sequence length	30	50
Number of temporal numerical features	75	50
Number of temporal categorical features	8	0
Number of static numerical features	3	3
Number of static categorical features	3	1

Table 2: The key properties of the two EHR datasets: MIMIC-III and eICU, used for EHR-Safe evaluation.

6 Supplementary Discussions: Additional Experiments

6.1 Propensity scores - Distinguishing synthetic data from original data

In this subsection, we train an ad-hoc binary classifier whose objective is to identify real samples from the synthetic samples. If the performance of the ad-hoc classifier (discriminator) is closer to 0.5 (random guessing), we can claim that the synthetic data preserve the original data properties well.

Models	MIMIC-III		eICU	
	Accuracy	AUC	Accuracy	AUC
GBDT	0.770	0.863	0.714	0.793
RF	0.805	0.884	0.784	0.858
GRU	0.764	0.852	0.877	0.954
LR	0.667	0.732	0.614	0.660
Average	0.751	0.832	0.747	0.816

Table 3: Propensity score performance with 4 different predictive models using MIMIC-III and eICU datasets. Performances are evaluated on original and synthetic test sets.

We also report the propensity scores per features to check which features are more realistic/unrealistic compared with the original features. As can be seen in Fig. 7, the discriminator performance is lower than 0.6 for most features.

6.2 Data coverage visualizations

Having similar coverage, and avoiding under-representation of certain data regimes, is crucial for synthetic data generation. We use t-SNE (t-distributed stochastic neighbor embedding) analyses to provide a qualitative intuition as to how well our synthetic data overlap the original data. More specifically, t-SNE analysis serves as a non-linear dimensionality reduction method to visualize high dimensional data by giving each data point a location in a two-dimensional map.

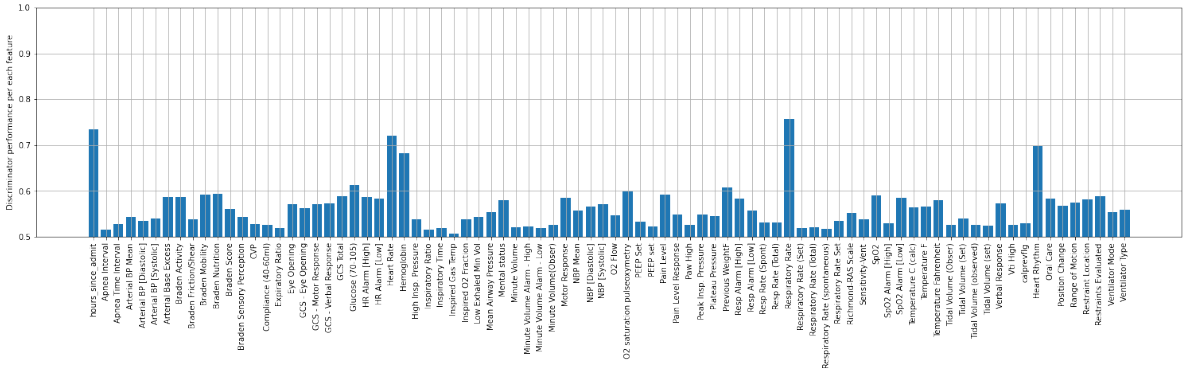

(a) Propensity scores of each feature on MIMIC-III dataset.

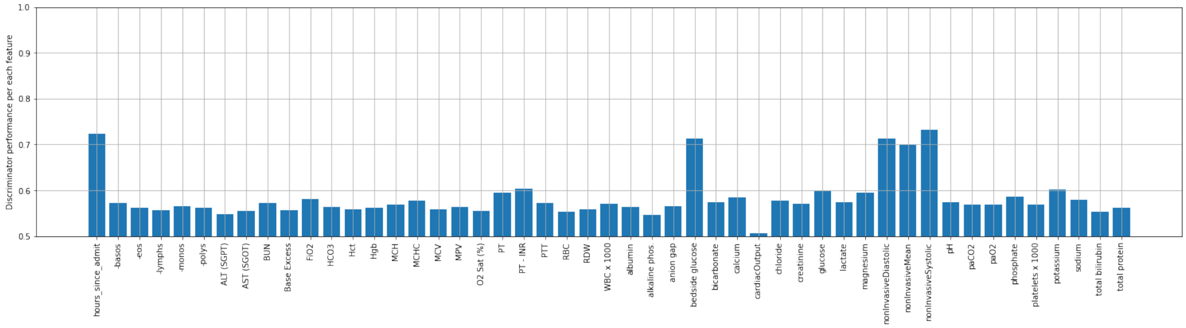

(b) Propensity scores of each feature on eICU dataset.

Figure 7: **Propensity score analyses per feature across two medical datasets** Note that the propensity scores of most features are less than 0.6 which is similar with random guessing.

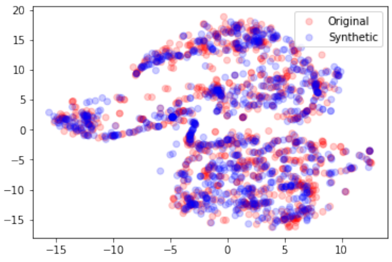

(a) MIMIC-III - Temporal data

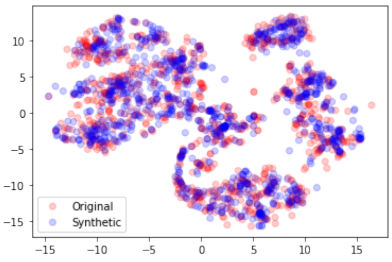

(b) MIMIC-III - Mask data

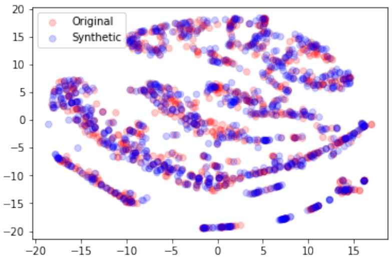

(c) MIMIC-III - Static data

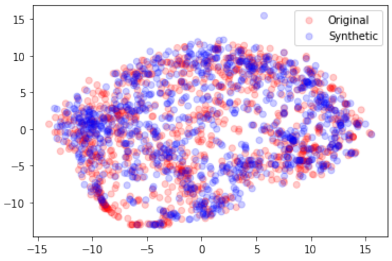

(d) eICU - Temporal data

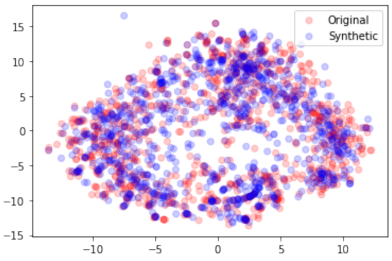

(e) eICU - Mask data

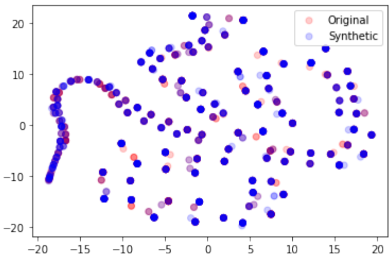

(f) eICU - Static data

Figure 8: **t-SNE analyses** Analyses on temporal, mask and static data on MIMIC-III and eICU datasets.

As Fig. 8 shows, the coverage of the synthetic data is very similar with the coverage of the original data. Note that for three-axes temporal and mask data, we first convert them into two axes data where each column represents each feature at each time point.

6.3 Algorithmic fairness analysis

In this subsection, we provide algorithmic fairness analyses for different sensitive attributes: gender, marital status, religion for MIMIC-III; and gender for eICU. We focus on the mortality prediction as the downstream task and random forest as the predictive model.

We utilize three different metrics to evaluate the algorithmic fairness of original and synthetic data:

- **Demographic parity**: Differences between probability of being assigned to the positive class, across the subgroups divided by the attributes;
- **Equalized odds**: True Positive Rates (TPR) and False Positive Rates (FPR) differences across the subgroups divided by the attributes;
- **Overall accuracy equality**: Performance (with AUC being the metric) differences across the subgroups divided by the attributes.

More details of these algorithmic fairness metrics can be found in [4]. Fig. 9 shows that the algorithmic fairness performances metrics between the original and synthetic data are consistent across various subgroups. In other words, the algorithmic fairness biases across different subgroups is not amplified by the synthetic data generated by EHR-Safe compared with original data.

6.4 Impacts of stochastic normalization

Fig. 10 shows the cumulative distribution function (CDF) curves with and without stochastic normalization, highlighting it's key role in improving the fidelity of synthetic data.

6.5 Additional Privacy Results

In this section, we present privacy attack results. For the preliminary results, we used the Euclidean distance metric for the kNN algorithms. However, since the data we generate is time series based type of the data, it is recommended to also consider distance metrics that cover time. We consider three more time-series distance metrics in kNN models from *tslearn* package:

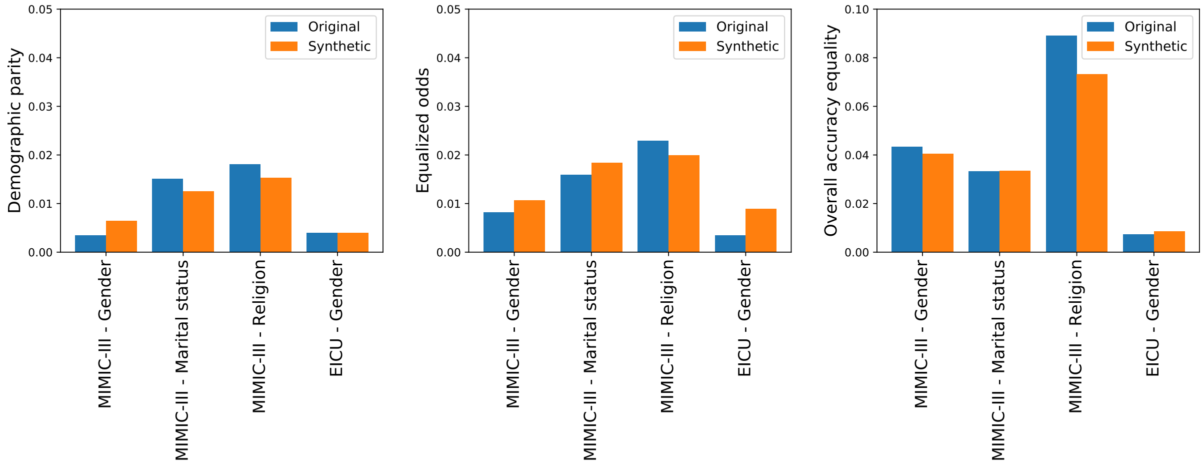

(a) Demographic parity (b) Equalized odds (c) Overall accuracy equality

Figure 9: **Algorithmic fairness analyses for multiple subgroups divided by sensitive attributes** In most cases, the algorithmic bias in the original data is inherited to the synthetic data, and not amplified.

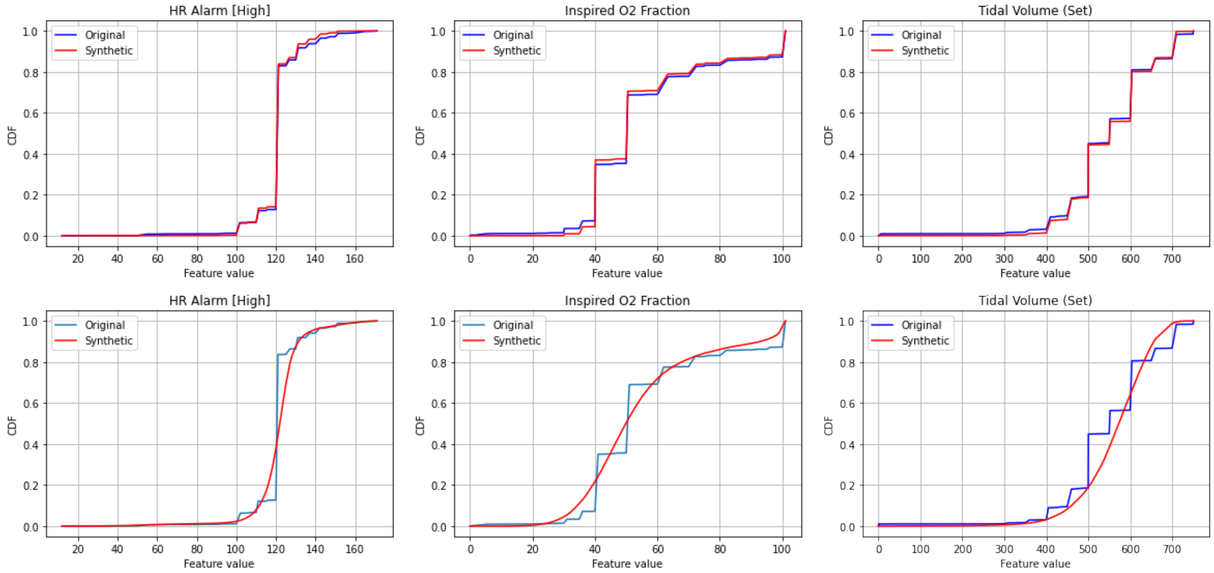

Figure 10: **CDFs with stochastic normalization (Upper) vs. without stochastic normalization (Lower)** Without stochastic normalization, it is very challenging for EHR-Safe to mimic the CDF of the original samples, especially for those with discrete jumps.

- Dynamic Time Wrapping (DTW): For measuring the distance between two temporal sequence that may have different speed.
- SoftDTW: A more advanced version of the DTW where the difference can be computed at every point. The implementation of this metric is much faster compared to DTW as well.
- Canonical Time Warping (CTW): An improved version of DTW where the difference can be calculated in more complex scenarios where there is rotation and transformation of the data over time.

The results are provided in Table 4.

Privacy metrics	Distance metrics	MIMIC-III		eICU	
		No privacy risk	EHR-Safe	No privacy risk	EHR-Safe
**Membership inference**	DTW	0.500	0.488	0.500	0.472
	SoftDTW	0.500	0.490	0.500	0.484
	CTW	0.500	0.469	0.500	0.461
**Re-identification**	DTW	0.050	0.054	0.064	0.079
	SoftDTW	0.048	0.057	0.066	0.078
	CTW	0.061	0.069	0.068	0.082
**Attribute-inference** Target - Gender	DTW	0.682	0.671	0.664	0.651
	SoftDTW	0.719	0.708	0.681	0.669
	CTW	0.723	0.714	0.692	0.685
**Attribute-inference** Target - Marital status	DTW	0.634	0.629	-	-
	SoftDTW	0.629	0.621	-	-
	CTW	0.638	0.631	-	-
**Attribute-inference** Target - Religion	DTW	0.627	0.620	-	-
	SoftDTW	0.632	0.619	-	-
	CTW	0.637	0.632	-	-

Table 4: Privacy risk evaluation with different distance metrics (DTW, SoftDTW, CTW). For membership inference, the ideal value is random guessing (i.e. 0.5) whether an original sample has been leveraged for training the synthetic data generation model. For the re-identification, the ideal case is to replace the synthetic data with holdout original data which is disjoint with the training data. For attribute inference attack, we set three static features (gender, race, medical status – note that eICU only has a gender attribute) as the specific attributes and report prediction AUC. The baseline scenario is measured by performing feature prediction using the original data. For multi-class data such as marital status or religion, we compute the pairwise AUC values across all possible categories and report their average values.

6.6 Statistical similarity

Fig. 11 shows the pairwise Pearson correlations - a measure of linear correlation between two sets of data to evaluate whether the correlation between features are well conserved - between temporal numerical features. We observe almost identical heatmaps indicating that the generated synthetic data largely conserve the original correlations. Table 5 and 6 present the statistical similarity per each feature in MIMIC-III and eICU datasets, respectively. Fig. 12 shows the top 10 frequent categories' frequencies for original and synthetic data. The distributions for the static and temporal categorical features are well aligned between original and synthetic data.

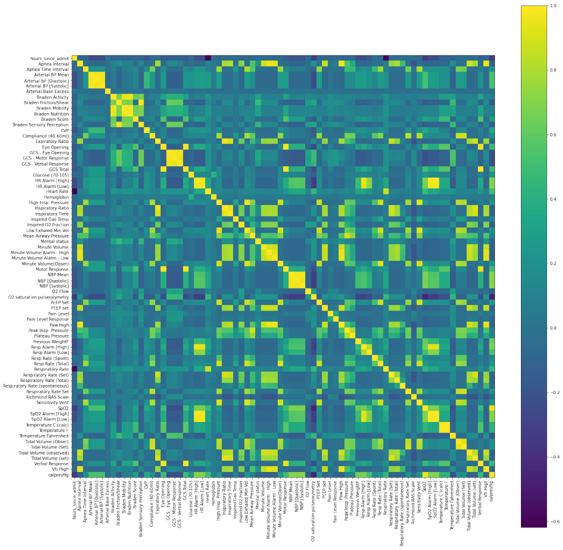

(a) MIMIC-III - Original data

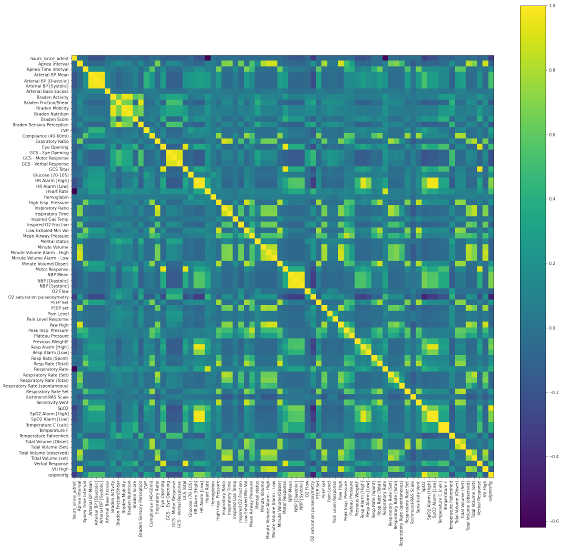

(b) MIMIC-III - Synthetic data

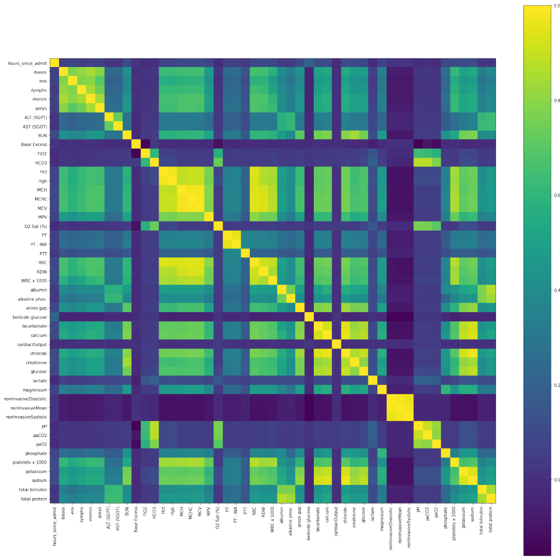

(c) eICU - Original data

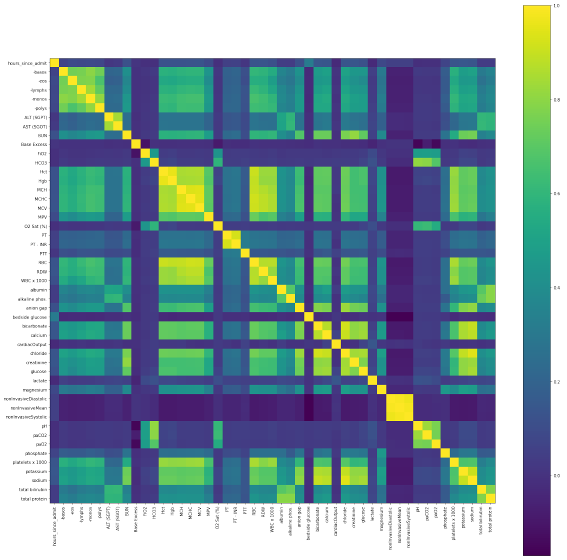

(d) eICU - Synthetic data

Figure 11: **Pearson correlation analyses** Analyses between temporal numerical features of original and synthetic data.

MIMIC-III Dataset							
Feature name	Original data			Synthetic data			KS-Stats
	Mean	Std	Miss rate (%)	Mean	Std	Miss rate (%)	
Arterial BP [Systolic]	116.93	24.12	70.63	118.42	20.76	70.00	0.0243
Arterial BP [Diastolic]	56.68	12.25	78.24	56.90	10.19	79.20	0.0318
Arterial BP Mean	77.66	14.43	78.26	76.97	12.83	79.29	0.0293
Eye Opening	3.29	1.08	78.60	3.41	1.01	79.90	0.052
Motor Response	5.39	1.31	79.43	5.23	1.48	79.43	0.0442
Verbal Response	3.41	1.85	79.48	3.45	1.88	79.44	0.0401
GCS Total	12.10	3.75	79.59	11.99	3.97	79.56	0.0412
Braden Activity	1.20	0.58	79.62	1.18	0.51	79.68	0.0117
Braden Mobility	2.55	0.73	79.96	2.54	0.67	80.19	0.0297
Braden Nutrition	2.26	0.63	80.03	2.28	0.62	80.32	0.0347
GCS - Eye Opening	3.33	1.04	81.62	3.51	0.93	83.07	0.0968
GCS - Motor Response	5.46	1.23	86.89	5.43	1.30	86.71	0.0103
GCS - Verbal Response	3.59	1.78	86.89	3.61	1.80	86.78	0.0191
Temperature F	98.07	3.05	87.03	98.06	1.32	86.76	0.0493
Temperature C (calc)	36.73	1.27	87.49	36.70	0.73	87.79	0.0486
Temperature Fahrenheit	97.87	3.30	87.54	97.89	1.25	88.27	0.046
CVP	10.38	4.74	87.55	10.05	3.77	88.15	0.0592
Pain Level	2.72	3.01	87.61	2.73	3.01	88.75	0.0148
Mean Airway Pressure	9.81	3.07	89.23	9.89	2.86	89.71	0.0265
Hemoglobin	10.46	1.77	89.23	10.47	1.72	89.74	0.0213
Glucose (70-105)	138.12	48.53	89.24	148.16	48.49	89.93	0.1003
Braden Sensory Perception	3.06	0.87	90.15	3.07	0.82	90.24	0.0174
Arterial Base Excess	-1.51	4.85	90.19	0.70	4.62	90.58	0.2262
Braden Friction/Shear	2.12	0.54	90.19	2.12	0.52	90.64	0.0159
Braden Score	14.45	2.34	90.66	14.51	2.18	91.87	0.0273
Peak Insp. Pressure	23.14	7.40	90.66	23.18	7.00	91.88	0.0173
Mental status	7.30	7.50	91.76	6.65	7.45	92.42	0.0435
O2 Flow	4.53	3.78	92.82	3.95	3.38	94.02	0.1055
Inspired O2 Fraction	55.17	20.93	93.30	59.11	21.65	94.24	0.0686
PEEP Set	5.64	1.89	94.11	5.70	1.74	94.82	0.0125
Resp Rate (Total)	17.42	5.18	94.19	17.15	4.82	95.77	0.0235
Richmond-RAS Scale	-0.71	1.59	94.31	-0.94	1.77	96.26	0.0521
Minute Volume(Obser)	8.90	2.55	94.49	9.11	2.54	94.48	0.0402
Low Exhaled Min Vol	4.28	1.10	94.50	3.96	0.76	95.31	0.1462
Sensitivity-Vent	2.22	0.46	94.51	2.20	0.43	94.75	0.0299
High Insp. Pressure	41.62	6.52	94.85	41.11	5.99	96.05	0.0516
Plateau Pressure	19.50	4.79	94.88	19.74	4.81	95.38	0.0336
Respiratory Rate Set	14.29	4.30	95.20	14.03	3.88	96.12	0.0574
Pain Level Response	1.00	1.62	95.73	1.23	1.72	96.81	0.0652
Tidal Volume (Obser)	553.14	104.53	96.17	574.78	97.72	96.06	0.0867
Tidal Volume (Set)	547.10	107.70	96.28	581.24	87.15	96.53	0.1358
PEEP set	5.71	2.02	96.39	5.67	1.70	96.75	0.0212
Resp Rate (Spont)	1.95	3.29	96.46	2.24	3.35	97.59	0.0503
Minute Volume	8.48	2.26	96.48	8.26	2.07	96.83	0.0555
Compliance (40-60ml)	29.41	8.77	96.63	29.18	8.88	97.30	0.0472
Tidal Volume (observed)	470.28	112.09	96.64	448.82	98.97	97.28	0.1143

Table 5: Distribution analyses on numerical temporal features of MIMIC-III data (top 16 - 51). KS-stats represents the maximum CDF difference between original and synthetic features (we ignore missing components when computing KS-stats).

Feature name	Original data			Synthetic data			KS-Stats
	Mean	Std	Miss rate (%)	Mean	Std	Miss rate (%)	
RBC	3.53	0.71	92.83	3.56	0.66	93.46	0.0256
MCHC	32.79	1.42	93.12	32.84	1.35	93.98	0.0256
MCV	90.18	6.22	93.14	90.41	5.75	93.79	0.0308
MCH	29.58	2.37	93.28	29.69	2.18	93.87	0.0197
RDW	15.42	2.24	93.38	15.31	2.22	94.01	0.0479
anion gap	10.50	4.08	93.48	10.22	3.76	93.98	0.0374
MPV	9.74	1.27	94.93	9.59	1.11	95.78	0.0639
lymphs	14.87	8.90	95.86	14.22	7.93	96.36	0.0311
monos	7.90	3.42	95.88	7.84	2.94	96.40	0.0373
eos	1.78	1.80	96.11	1.63	1.63	96.61	0.0251
magnesium	1.94	0.30	96.31	1.95	0.27	97.32	0.0362
basos	0.27	0.37	96.33	0.23	0.35	96.94	0.0488
polys	71.66	14.09	96.35	73.06	11.08	96.78	0.0428
albumin	2.79	0.67	97.04	2.87	0.65	97.59	0.0539
AST (SGOT)	46.48	50.48	97.51	42.97	48.16	97.90	0.0892
total protein	6.05	0.92	97.51	6.18	0.83	97.98	0.0750
ALT (SGPT)	43.96	48.03	97.53	40.78	45.94	97.99	0.0709
alkaline phos.	97.72	50.62	97.54	97.04	45.32	97.99	0.0472
total bilirubin	0.83	0.77	97.63	0.87	0.70	98.04	0.0577
PT - INR	1.52	0.58	97.64	1.60	0.64	98.56	0.0504
PT	17.28	5.70	97.73	17.83	6.04	98.62	0.0389
phosphate	3.25	1.07	97.77	3.35	0.96	98.63	0.0671
PTT	42.06	17.44	98.55	45.37	19.72	99.20	0.0806
paO2	110.67	57.50	98.59	115.28	60.21	99.25	0.0439
pH	7.34	0.12	98.60	7.31	0.14	99.22	0.0934
paCO2	40.68	9.66	98.61	42.01	10.35	99.22	0.0643
HCO3	22.87	5.39	98.66	23.04	5.93	99.23	0.0489
FiO2	47.04	28.76	98.77	47.71	30.41	99.32	0.0428
O2 Sat (%)	94.99	7.51	98.88	94.61	8.45	99.30	0.0412
Base Excess	-1.96	6.13	98.96	-2.32	6.64	99.40	0.0391
lactate	1.92	1.17	99.16	2.68	1.76	99.45	0.2411
cardiac Output	3.28	2.12	99.83	4.14	1.93	99.91	0.2819

Table 6: Distribution analyses on numerical temporal features of eICU data (top 16 - 47). KS-stats represents the maximum CDF difference between original and synthetic features (we ignore missing components when computing KS-stats).

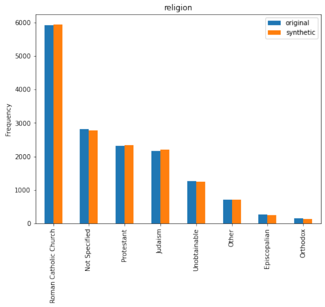

(a) Religion

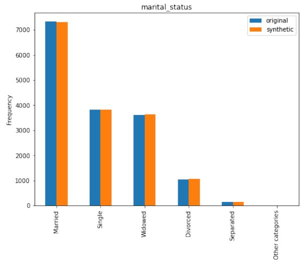

(b) Marital status

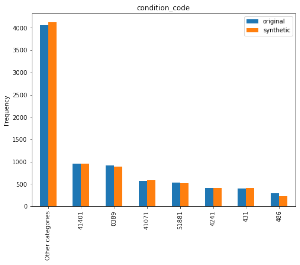

(c) Medical code

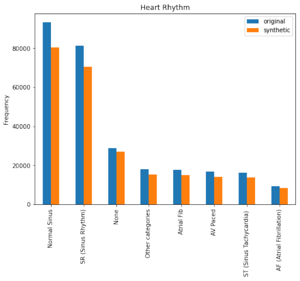

(d) Heart Rhythm

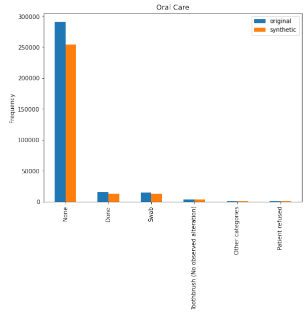

(e) Oral Care

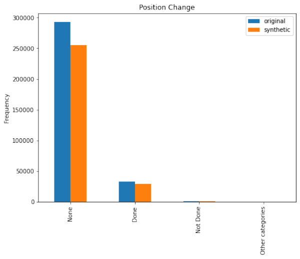

(f) Position Change

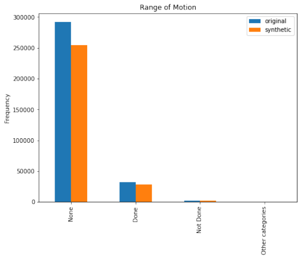

(g) Range of Motion

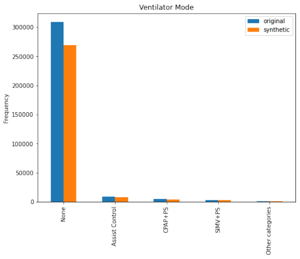

(h) Ventilator Mode

Figure 12: **Distribution analyses** Analyses on static and temporal categorical features on MIMIC-III datasets.

6.7 Feature importance analyses

In this section, we introduce feature importance comparisons as another fidelity measure to verify that the synthetic data can preserve the important feature characteristics of the original dataset. More specifically, we extracted the feature importance (computed by mean decrease in impurity (`https://scikit-learn.org/stable/auto_examples/ensemble/plot_forest_importances.html#feature-importance-based-on-mean-decrease-in-impurity`)) of two models: (i) trained on original data, (ii) trained on synthetic data using Random Forest (RF) and Gradient Boosting Decision Trees (GBDT) methods. Then, we plot top-30 ranked important features to qualitatively compare their similarities. As can be seen in Fig. 13, the feature importance of the two models (trained on real vs trained on synthetic) is highly similar, verifying that the synthetic data preserves the feature importance of the original data. For instance, 80% of top 10 important features are overlapped between original and synthetic data for both MIMIC-III and eICU datasets.

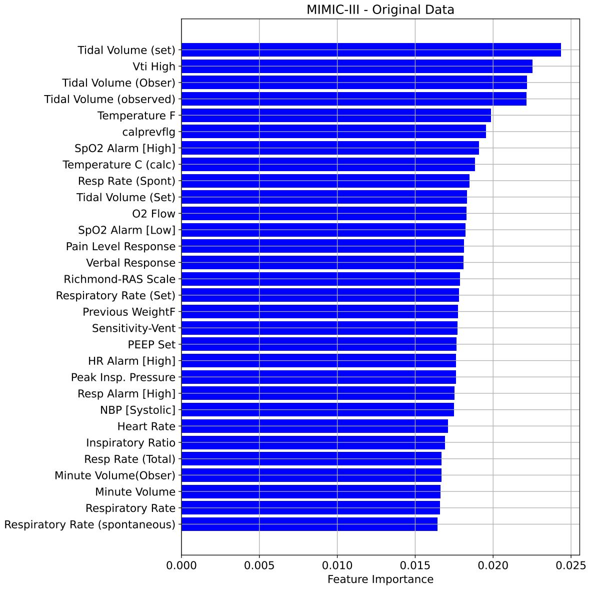

(a) MIMIC-III: Top-30 important features discovered by RF using original data.

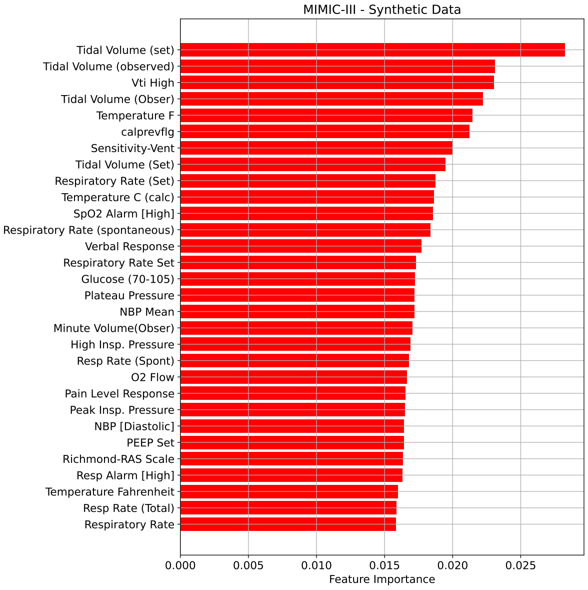

(b) MIMIC-III: Top-30 important features discovered by RF using synthetic data.

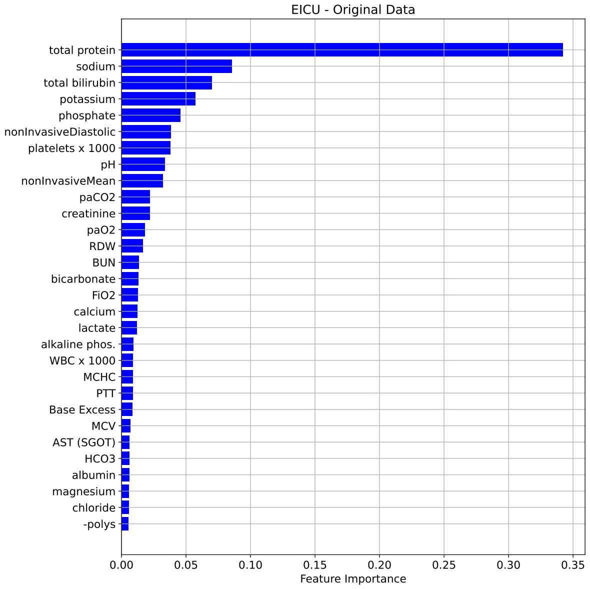

(c) eICU: Top-30 important features discovered by GBDT using original data.

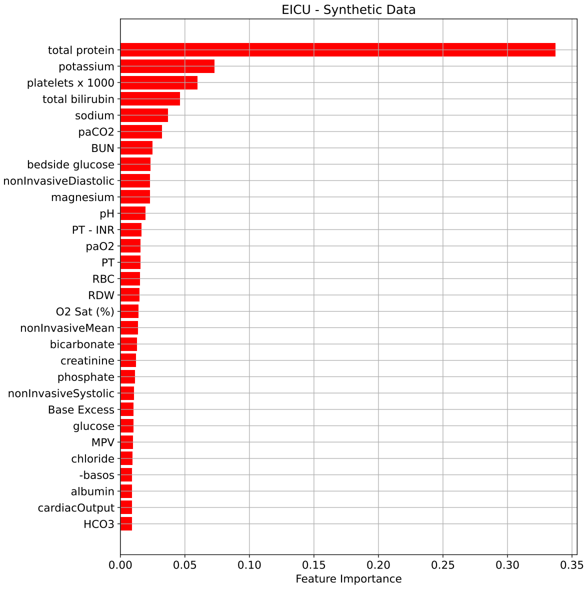

(d) eICU: Top-30 important features discovered by GBDT using synthetic data.

Figure 13: **Feature importance for downstream tasks** (left) original (right) synthetic data discovered by Random Forest (RF) and Gradient Boosting Decison Trees (GBDT).

7 Supplementary Discussions: Additional examples of real and synthetic EHR data

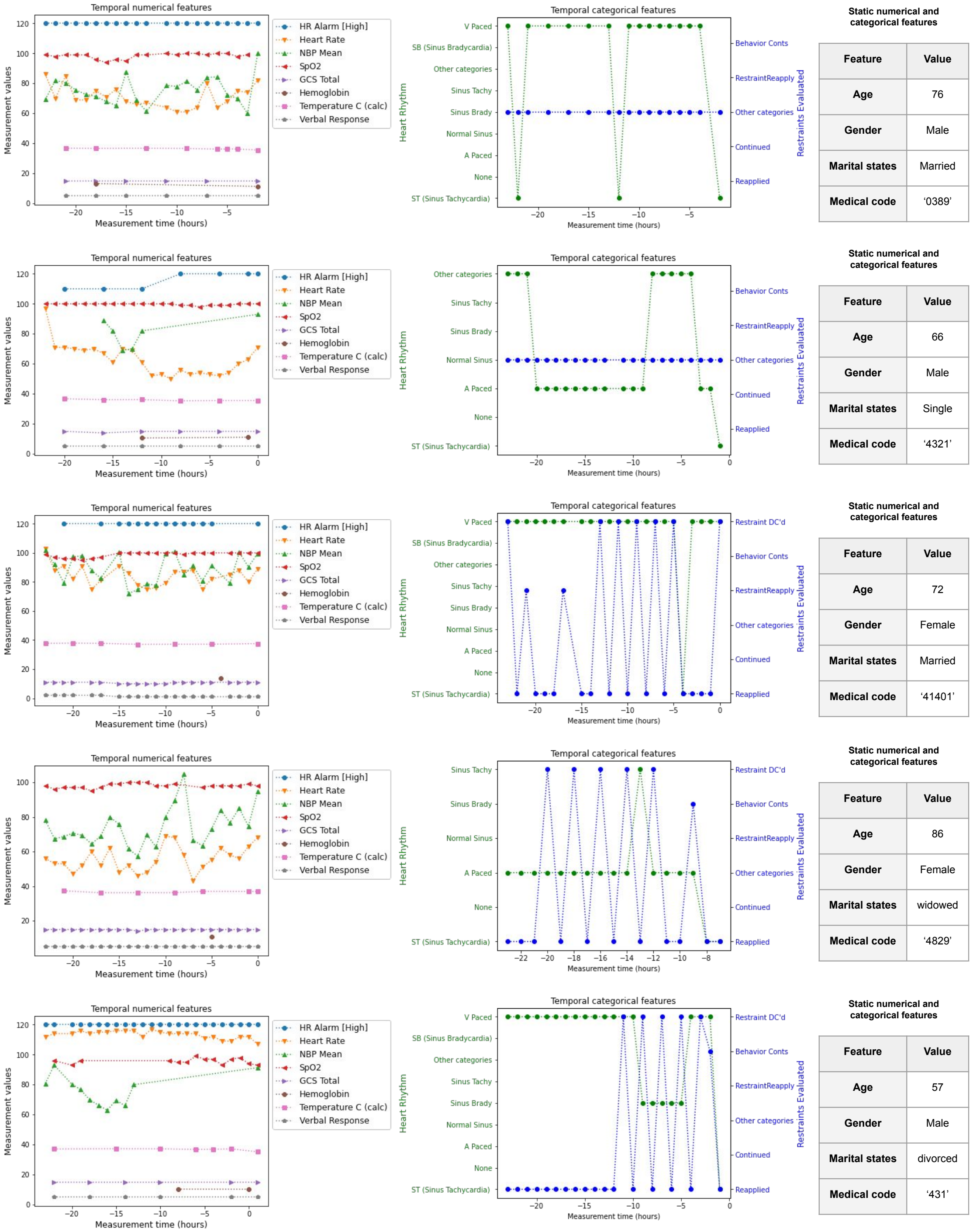

Feature	Value
Age	76
Gender	Male
Marital states	Married
Medical code	'0389'

Feature	Value
Age	66
Gender	Male
Marital states	Single
Medical code	'4321'

Feature	Value
Age	72
Gender	Female
Marital states	Married
Medical code	'41401'

Feature	Value
Age	86
Gender	Female
Marital states	widowed
Medical code	'4829'

Feature	Value
Age	57
Gender	Male
Marital states	divorced
Medical code	'431'

Figure 14: **Additional example of real EHR data examples** They contains static and temporal features (both numerical and categorical).

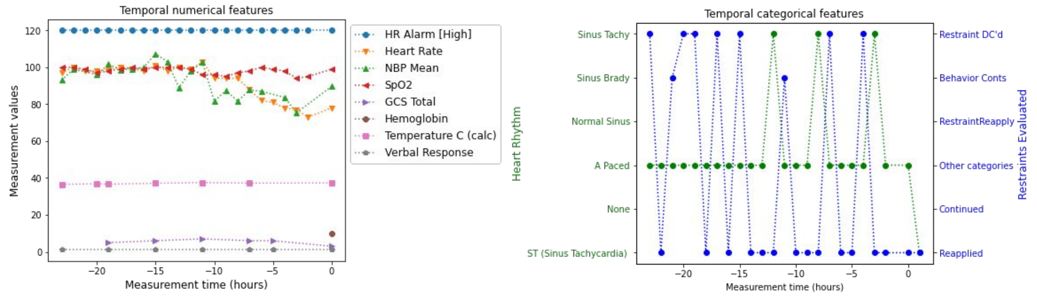

Static numerical and categorical features

Feature	Value
Age	51
Gender	Male
Marital states	Single
Medical code	'5770'

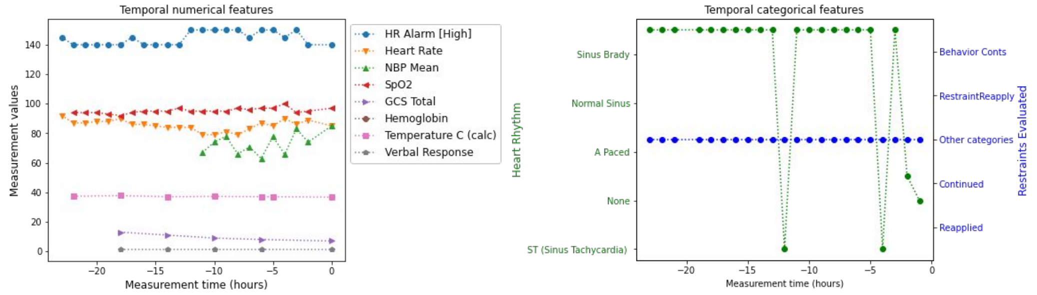

Static numerical and categorical features

Feature	Value
Age	73
Gender	Male
Marital states	Married
Medical code	'0845'

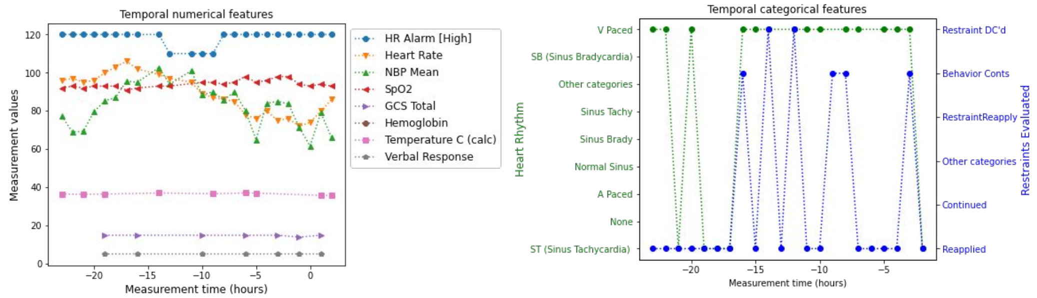

Static numerical and categorical features

Feature	Value
Age	39
Gender	Female
Marital states	divorced
Medical code	'99859'

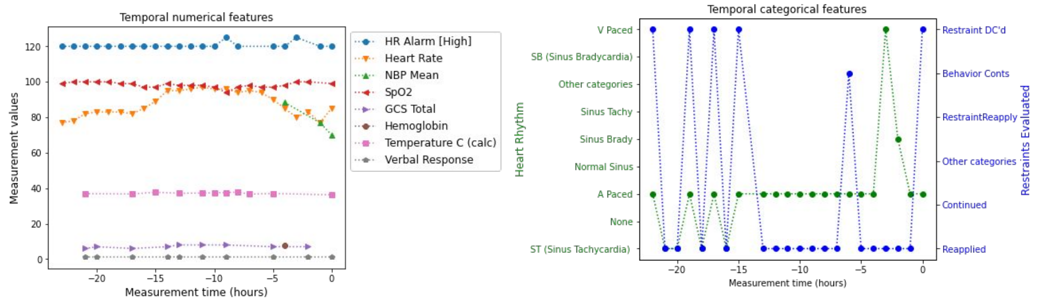

Static numerical and categorical features

Feature	Value
Age	83
Gender	Female
Marital states	Married
Medical code	'5070'

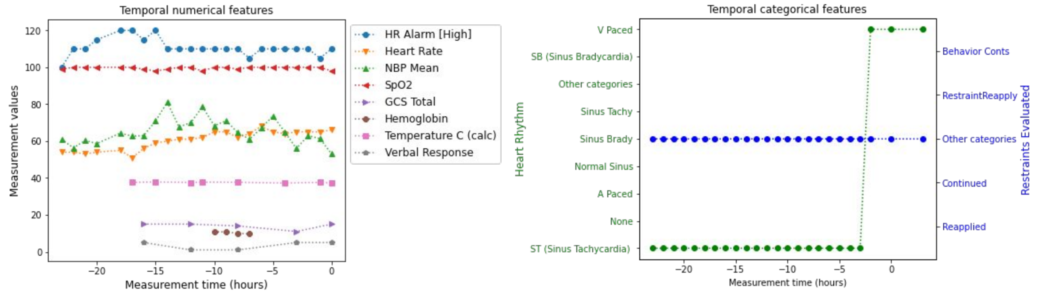

Static numerical and categorical features

Feature	Value
Age	73
Gender	Male
Marital states	Married
Medical code	'2761'

Figure 15: **Additional example of synthetic EHR data examples** They contains static and temporal features (both numerical and categorical).

References

[1] C. Esteban, S. L. Hyland, and G. Rätsch, "Real-valued (medical) time series generation with recurrent conditional gans," *Preprint at https://arxiv.org/abs/1706.02633*, 2017.

[2] J. Yoon, D. Jarrett, and M. Van der Schaar, "Time-series generative adversarial networks," *Advances in neural information processing systems*, vol. 32, 2019.

[3] O. Mogren, "C-rnn-gan: Continuous recurrent neural networks with adversarial training," *Preprint at https://arxiv.org/abs/1611.09904*, 2016.

[4] N. Mehrabi, F. Morstatter, N. Saxena, K. Lerman, and A. Galstyan, "A survey on bias and fairness in machine learning," *ACM Computing Surveys (CSUR)*, vol. 54, no. 6, pp. 1–35, 2021.
